# Photodynamic enhancement of PROTAC prodrug activation in hypoxic tumors

**DOI:** 10.1016/j.apsb.2025.07.020

**Published:** 2025-07-14

**Authors:** Zhongliang Fu, Chunrong Yang, Yuchen Yang, Meichen Pan, Hongwei Hou, Jinghong Li

**Affiliations:** a School of Biomedical Sciences, Hunan University, Changsha 410082, China; b Beijing Life Science Academy, Beijing 102209, China; c New Cornerstone Science Laboratory, Department of Chemistry, Key Laboratory of Bioorganic Phosphorus Chemistry & Chemical Biology, Tsinghua University, Beijing 100084, China; d Center for BioAnalytical Chemistry, Hefei National Laboratory of Physical Science at Microscale, University of Science and Technology of China, Hefei 230026, China

**Keywords:** PROTACs, Hypoxia-activated, Prodrugs, Photodynamic therapy, Tumors, Fluorescence imaging, Protein degradation, Near-infrared laser irradiation

## Abstract

Proteolysis-targeting chimeras (PROTACs) have emerged as a promising therapeutic strategy for targeted protein degradation. However, the clinical application of PROTACs may be hindered by off-target toxicity resulting from non-tissue-specific protein degradation and ingenious prodrug strategies may open new avenues to addressing this concern. Herein, we propose a light-induced positive feedback strategy to use photodynamic therapy (PDT) to improve the activation efficiency of PROTAC prodrugs, monitor PROTAC release, and combine PROTAC to induce tumor cell apoptosis. In the hypoxic tumor microenvironment, the azo bond in AZO-PRO selectively cleaves, triggering the release of the potent protein degrader PRO and the multifunctional photosensitizer. Once activated, the fluoresce signal of the photosensitizer dramatically recovers, allowing monitoring of prodrug activation. Additionally, upon irradicating the tumor site using near-infrared (NIR) laser, PDT exacerbates tumor hypoxia, further promoting AZO-PRO activation. Our work introduces a novel approach to efficiently track and activate PROTAC prodrugs, enhance their antitumor efficacy, and mitigate off-target systemic toxicity.

## Graphical abstract

This study proposed a light-induced positive feedback strategy, using PDT to aggravate tumor hypoxia to promote hypoxia-responsive prodrug activation, and provide real-time feedback on prodrug activation through fluorescence recovery.

**Figure:**
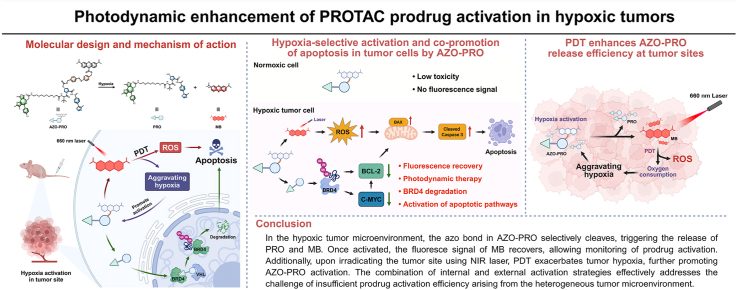

## 1 Introduction

Proteolysis-targeting chimera (PROTAC) is an emerging therapeutic strategy for targeted protein degradation that enables the selective and targeted elimination of proteins^1^^,^^2^. PROTAC is formed by an E3 ligase ligand, a protein of interest (POI) binding ligand, and a chemical linker that connects the ligands. By simultaneously recruiting an E3 ligase and a POI, PROTACs facilitate the formation of ternary complexes, which further promote the polyubiquitination and degradation of POI through the ubiquitin-proteasome system^3^^,^^4^. This unique mechanism not only provides an alternative approach to targeting “undruggable” proteins but also offers several advantages over traditional small-molecule inhibitors, such as catalytic properties, reduced dosage, and improved efficacy against resistance mechanisms^5^^,^^6^. However, the limited selectivity between cancer cells and normal cells may lead to systemic toxicity, impeding their clinical application^7^, ^8^, ^9^. Prodrug strategies have been shown to improve selectivity, extend pharmacokinetics, reduce off-target toxicity, and enhance therapeutic efficacy in clinical practice^10^. Several stimuli-responsive PROTACs have been developed for tumor-specific protein degradation through both endogenous biomarkers (*e.g*., reactive oxygen species^11^ and overexpressed enzymes^12^^,^^13^) and external stimuli (*e.g*., light^14^^,^^15^ and X-ray^16^). These activatable PROTACs demonstrate significant potential in controlling protein degradation.

Hypoxia, a prominent characteristic of malignant tumors, provides a promising avenue for the development of hypoxia-responsive prodrugs^17^, ^18^, ^19^, ^20^. Due to the rapid proliferation of tumor cells and the insufficient oxygen supply, hypoxic solid tumors exhibit overexpression of certain reducing substances, such as nitro reductase (NTR) ^21^^,^^22^ and azo reductase^23^^,^^24^. To date, several NTR-activated PROTAC prodrugs have been developed. For instance, Shi et al.^25^ developed NTR-responsive PROTACs by incorporating nitroimidazole moieties onto the von Hippel-Lindau (VHL) E3 ubiquitin ligase ligand. These PROTACs remain inactive in normal tissues but are activated by NTR overexpression in tumor tissues. Similarly, Do et al. ^26^ designed an enzyme clickprotacs (ENCTACs) with a CRBN-binding ligand that becomes active under hypoxic conditions *via* NTR and glutathione, facilitating the degradation of BRD4. Although these PROTAC prodrugs show potential against tumors, the nitroimidazole masking group may result in limited hypoxic selectivity and undesired toxicity in healthy tissues^27^. Additionally, the heterogeneous distribution of hypoxia in solid tumors often limits the effectiveness of hypoxia-responsive prodrugs, resulting in suboptimal therapeutic outcomes in some clinical trials^28^, ^29^, ^30^, ^31^. Therefore, the development of more intelligent hypoxic activation strategies to improve tissue specificity and promote effective prodrug activation remains a significant challenge in PROTAC design.

Photodynamic therapy (PDT), a promising modality for cancer treatment, has gained clinical approval for various diseases due to its high spatiotemporal selectivity and minimal invasiveness^32^^,^^33^. PDT destroys tumor cells mainly by activating photosensitizers to generate cytotoxic reactive oxygen species (ROS), a process accompanied by sustained oxygen consumption and vascular damage^34^, ^35^, ^36^. Additionally, PDT-induced hypoxia can enhance the release of hypoxia-responsive prodrugs. Methylene blue (MB), an FDA-approved drug for treating malaria and methemoglobinemia, has also been shown to be an effective photosensitizer for PDT^37^^,^^38^.

**Figure 1:**
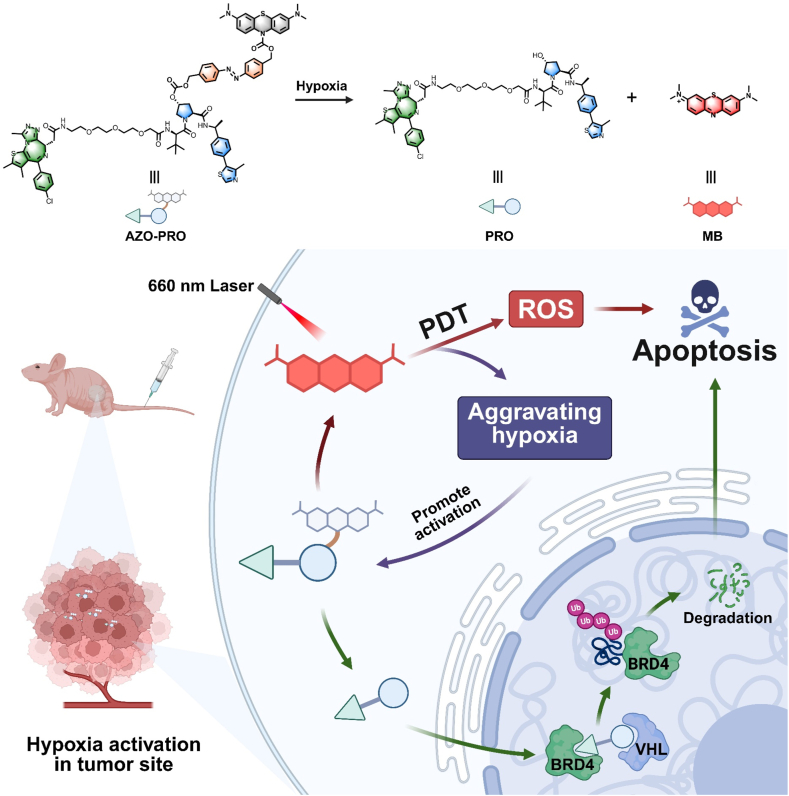
Schematic diagram of PDT enhancing the activation of hypoxia-responsive PROTAC prodrug and inducing tumor cell apoptosis in combination. After reaching the hypoxic tumor microenvironment, the prodrug is activated to release PROTAC (PRO) and photosensitizer (MB). The released PRO selectively induces BRD4 protein degradation through the proteasome mechanism. MB is irradiated with NIR laser to effectively consume oxygen through the PDT process, and the more hypoxic tumor site further promotes the activation of the prodrug through positive feedback. In addition, the ROS released by MB synergizes with PRO to induce tumor cell apoptosis.

In this study, we proposed a light-induced positive feedback strategy, using PDT to aggravate tumor hypoxia to promote hypoxia-responsive prodrug activation, and provide real-time feedback on prodrug activation through fluorescence recovery. At the same time, PDT and PROTAC molecules are combined to enhance the therapeutic effect of hypoxic tumor treatment. We designed a PROTAC prodrug (AZO-PRO) that links MB and a PROTAC moiety (PRO) through a hypoxia-activated azobenzene structure, and confirmed the synergistic antitumor effects of PDT and the activated PROTAC (Fig. 1). The azo bond is cleaved in the hypoxic microenvironment, releasing both MB and PRO, which monitor drug release and degrade bromodomain and extraterminal domain (BET) proteins, respectively. The degradation inhibits BET protein-mediated transcriptional regulation, upregulates the pro-apoptotic Bcl-2-associated X (BAX) gene, and promotes tumor cell apoptosis. Additionally, the released MB enhances tumor hypoxia *via* the PDT process under 660 nm laser irradiation, further promoting PRO release. *In vivo* fluorescence monitoring revealed that AZO-PRO accumulates at the tumor site and is activated by the hypoxic tumor microenvironment within 24 h. Upon precise irradiation of the tumor site, the fluorescence signal increases further, indicating that PDT enhances AZO-PRO activation. Moreover, *in vivo* experiments demonstrated that combining PDT with AZO-PRO significantly enhances therapeutic efficacy, offering a strategy to improve PROTAC drug release and synergistic antitumor effects.

## 2 Materials and methods

The company and catalog numbers of commercial reagents are provided in the Materials section of Supporting Information.

### 2.1 Spectral test

Ultraviolet (UV) absorption and fluorescence spectra. Appropriate amounts of AZO-PRO were weighed and dissolved with DMSO to make a 10 mmol/L stock solution and stored at −80 °C in aliquots. The solvent was bubbled with argon for 1 h to remove oxygen from it. AZO-PRO (50 μmol/L) was mixed with Na_2_S_2_O_4_ (50 mmol/L) in PBS buffer solution (5 mmol/L, *V*_Acetonitrile_: *V*_PBS_ = 1:1) at 37 °C, UV and fluorescence spectra were studied with a UV–visible spectrophotometer and fluorometer at different time points.

Selectivity experiment. The solvent was bubbled with argon for 1 h to remove oxygen from it. AZO-PRO (50 μmol/L) was mixed with Na_2_S_2_O_4_ (12.5 mmol/L), Arginine (5 mmol/L), Glutamate (5 mmol/L), Cysteine (5 mmol/L), NADPH (0.5 mmol/L), KCl (5 mmol/L), NaCl (5 mmol/L), and CaCl_2_ (5 mmol/L), MgCl_2_ (5 mmol/L), NaClO (5 mmol/L), H_2_O_2_ (5 mmol/L), glucose (5 mmol/L), Glutathine (5 mmol/L), Vitamin C (5 mmol/L), or H_2_O (Blank) at 37 °C and subjected to fluorescence spectroscopy after 4 h of reaction.

### 2.2 HPLC analysis

HPLC conditions. Welch Ultisil XB-C18, 5 μm, 4.6 mm × 250 mm column, flow rate of 0.5 mL/min, detection wavelength: 254 nm; analytical methods: gradient elution with 30% acetonitrile–water solution (containing 0.1% trifluoroacetic acid) was performed at 0–3 min, followed by gradient elution with 30%–95% acetonitrile–water solution (containing 0.1% trifluoroacetic acid) at 3–13 min, followed by continued elution with 95% acetonitrile–water solution for 5 min, followed by gradient elution with 95%–30% acetonitrile–water solution (containing 0.1% trifluoroacetic acid), the detection was stopped in 30 min. All solvents used in experiments were of HPLC grade.

Release test. The solvent was bubbled with argon for 1 h to remove oxygen. AZO-PRO (100 μmol/L) was mixed with Na_2_S_2_O_4_ (50 mmol/L) and reacted at room temperature for 2 h. The reaction solution was then analyzed by HPLC and ultra-high resolution mass spectrometry.

Stability test. DMEM complete medium: add fetal bovine serum (FBS) and penicillin–streptomycin mixture to DMEM high glucose medium to prepare DMEM complete medium containing 10% FBS and 1% penicillin–streptomycin mixture. Dilute AZO-PRO with an initial concentration of 10 mmol/L to a final concentration of 100 μmol/L with PBS, DMEM complete medium or FBS solution containing 1% (*v*/*v*) DMSO. Place at room temperature for different time periods (0, 12, 24, 36, 48, 60, 72 h) and detect by HPLC.

### 2.3 Singlet oxygen measurement

The solvent was bubbled with argon for 1 h to remove oxygen from it. AZO-PRO (20 μmol/L) was mixed with Na_2_S_2_O_4_ (20 mmol/L) and reacted at room temperature for 2 h to obtain the test solution. Subsequently, an acetonitrile solution containing AZO-PRO, AZO-PRO + Na_2_S_2_O_4_, acetonitrile (control), and DPBF (60 μmol/L) was thoroughly mixed and subjected to irradiation from a 660 nm laser (15 mW/cm^2^) for varying time intervals. The absorbance at 411 nm was then measured.

### 2.4 Cell culture

MCF-7 cells were purchased from Wuhan Pricella Biotechnology Co., Ltd. The cells were cultured in Dulbecco's modified Eagle's medium (DMEM) supplemented with 10% fetal bovine serum (Gibco) and 1% penicillin–streptomycin (YEASEN, Shanghai, China). The cells were maintained at a temperature of 37 °C with a CO_2_ concentration of 5% in a humidified atmosphere. All cell experiments under hypoxic conditions were performed in a 2.5 L sealed culture tank (Mitsubishi Gas Chemical Company, C-31) equipped with an anaerobic gas generating bag (Mitsubishi Gas Chemical Company, C-1), and an oxygen indicator (Mitsubishi Gas Chemical Company, C-22) was used to determine that the oxygen concentration in the culture tank was always below 1%.

### 2.5 Cell viability assays

MCF-7 cells were seeded in 96-well plates at a density of 5000 cells per well and adhered to the wall at 37 °C for 24 h. The cells were divided into four groups: PRO (Normoxia), AZO-PRO (Normoxia), AZO-PRO (Hypoxia), and AZO-PRO + laser (Hypoxia). Different concentrations of PRO and AZO-PRO were added to the well plates, and the PRO (Normoxia) and AZO-PRO (Normoxia) groups were incubated under normoxia for 72 h; the AZO-PRO (Hypoxia) group was incubated in a hypoxic bag for 12 h and then incubated under normoxia for 60 h; the AZO-PRO + laser (Hypoxia) group was incubated in a hypoxic bag for 12 h, and then irradiated with a 660 nm laser at a power density of 0.8 W/cm^2^ for 5 min, and then incubated under normoxia for 60 h. The cell viability was measured with a Promega CellTiter 96® kit according to the manufacturer's instructions. Luminescence was measured by a plate reader (EnVision, Perkin). Data are analyzed with GraphPad Prism software to obtain the IC_50_ values of each test compound.

### 2.6 Confocal fluorescence imaging

Activation of prodrug in cells. MCF-7 cells were cultured in confocal dishes for 24 h, then AZO-PRO (5 μmol/L) was added, and the cells were incubated for 12 h under normoxic or hypoxic conditions. Fluorescence imaging was subsequently performed with confocal microscopy. Ex = 660 nm, Em = 680–740 nm.

Intracellular singlet oxygen detection. The MCF-7 cells were cultured in confocal petri dishes for 24 h, followed by the addition of AZO-PRO (5 μmol/L) to the dishes. Subsequently, the cells were further cultured for an additional 12 h under normoxic or hypoxic conditions. The material was then aspirated, followed by the addition of the DCFH-DA probe (10 μmol/L) and incubation at 37 °C for 20 min. Subsequently, the cells were washed with serum-free medium. The cells were then divided into two groups: one group was irradiated with 660 nm laser (0.8 W/cm^2^, 5 min), and the other group was not irradiated. The treated cells were imaged by confocal imaging. DCFH-DA probe: Ex = 488 nm, Em = 520–600 nm.

Live/dead cell staining assay. The MCF-7 cells cultured in the confocal culture dishes were divided into 6 groups: PBS (Normoxia), PBS (Hypoxia), AZO-PRO (Normoxia), AZO-PRO (Hypoxia), PRO (Normoxia), and AZO-PRO + Laser (Hypoxia) group. Among them, MCF-7 cells in the normoxia group were incubated with different materials (400 nmol/L) and then washed twice with PBS after 48 h of cultivation under normoxic conditions. MCF-7 cells in the hypoxia group were incubated with different materials (400 nmol/L) and then cultured in a hypoxic bag for 12 h. After that, they were treated with or without 660 nm laser (0.8 W/cm^2^) for 5 min, followed by continued cultivation under normoxic conditions for 36 h and subsequent washing twice with PBS. Then the cells were stained with Calcein AM (1000 ×) and PI (1000 ×) solutions for 20 min, washed with PBS by 3 times, and confocal fluorescence imaging. Calcein AM: Ex = 494 nm, Em = 505–525 nm. PI: Ex = 535 nm, Em = 600–630 nm.

Cell immunofluorescence staining. Glass coverslips (NEST 15 mm diameter) were placed in 12-well plates. 1 × 10^5^ MCF-7 cells were seeded in each well and cultured for 24 h for cell attachment. The cells were then treated with 0.1% DMSO, 100 nmol/L PRO or 100 nmol/L AZO-PRO. The normoxia group was incubated in an incubator for 24 h, while the hypoxia group was subjected to a 12-h incubation in a hypoxia bag followed by an additional 12-h exposure to normoxic conditions. After 12 h of hypoxia treatment, the cells in laser group were irradiated with 660 nm laser at a power density of 0.8W/cm^2^ for 5 min, and then cultured in normoxia for 12 h. The coverslips were washed three times with ice-cold PBS, followed by fixation in 4% paraformaldehyde, permeation with Triton X-100, blocking with 5% BSA for 1 h, anti-BRD4 (1:100, Abcam, ab128874, UK) immunostaining for 2 h, Alexa Fluor 488 labeled goat anti-rabbit secondary antibody (1:500, Beyotime, A0423, Shanghai, China) and DAPI staining. Fluorescent images were taken by a Leica confocal microscope. Alexa Fluor 488: Ex = 488 nm, Em = 510–560 nm. DAPI: Ex = 405 nm, Em = 415–465 nm.

Mitochondrial membrane potential (MMP) assay under normoxic and hypoxic Conditions.The MCF-7 cells cultured in the confocal culture dishes were divided into 6 groups: PBS (Normoxia), PBS (Hypoxia), AZO-PRO (Normoxia), AZO-PRO (Hypoxia), PRO (Normoxia), and AZO-PRO + Laser (Hypoxia) group. Among them, MCF-7 cells in the normoxia group were incubated with different materials (400 nmol/L) and then washed with PBS after 24 h of cultivation under normoxic conditions. MCF-7 cells in the hypoxia group were incubated with different materials (400 nmol/L) and then cultured in a hypoxic bag for 12 h. After that, they were treated with or without 660 nm laser (0.8 W/cm^2^) for 5 min, followed by continued cultivation under normoxic conditions for 12 h and subsequent washing with PBS. MCF-7 cells were incubated with JC-1 staining solution (Beyotime Institute of Biotechnology, C2005, Shanghai, China) following the manufacturer's protocol. The cells were then photographed using Confocal fluorescence microscopy in the red channel for J-aggregates and green channel for J-monomer, separately. Flow cytometry was exerted by digestion of MCF-7 cells from the 12 well plate and treated with similar manipulation as stated before.

### 2.7 Apoptosis detection

MCF-7 cells were seeded in 12 well plate at a density of 2 × 10^5^ cells per well and adhered for 24 h at 37 °C. The cells were treated with DMSO, MB, PRO or AZO-PRO (400 nmol/L). The normoxia groups were cultured in an incubator for 48 h, and the hypoxia groups were incubated in a hypoxia bag for 12 h, and then incubated under normoxia conditions for another 36 h. The laser groups were irradiated with a 660 nm laser at a power density of 0.8 W/cm^2^ for 5 min after 12 h hypoxic treatment. Cells were washed three times with precooled PBS and subsequently digested with 0.25% trypsin without EDTA at 37 °C and made into a cell suspension solution. The supernatant was discarded after centrifugation at 215×*g* for 5 min. Cells were resuspended using PBS precooled at 4 °C and centrifuged again under the same conditions, and the supernatant was discarded. The staining procedure was subsequently performed according to the Annexin V-FITC/PI apoptosis detection kit instructions. The 1 × 10^6^ cells were resuspended in 100 μL binding buffer, mixed with 5 μL Annexin V-FITC solution and incubated for 5 min. Then 5 μL propidium iodide solution was added and PBS (200 μL) was mixed, and immediately detected by flow cytometry.

### 2.8 Western blotting

MCF-7 cells were seeded at 5 × 10^5^ cells per well in six-well plates for 24 h before treatment. Cells were scraped off with a spatula after treatment with PRO or AZO-PRO for 24 h. For the normoxia group, the cells were cultured in incubator for 24 h after the addition of the compound. For the hypoxia group, the cells were cultured in anoxic bags for 12 h after the addition of the compound, followed by irradiation with or without a 660 nm laser, and then continued to culture under normal oxygen conditions for 12 h. For cell lysis, cells were washed with ice-cold PBS and lysed in ice-cold RIPA lysis buffer (containing 1% protease and phosphatase inhibitors). Lysates were violent vortexed and cleared by centrifugation at 18,756×*g* for 15 min at 4 °C. Protein concentration was measured by BCA Protein Assay Kit (Epizyme, USA). The proteins were resolved in 4%–12% Bis-Tris SurePAGE gel (Genscript, USA) and transferred to 0.45 μm PVDF membrane (Merck Millipore, Germany) for Western blotting. The membrane was blocked by QuickBlock™ Western blocking buffer (Beyotime, Shanghai, China) for 20 min at room temperature. Blots were incubated in anti-BRD4 antibody (1:1000; Abcam, ab128874, UK), anti-c-Myc antibody (1:1000; Abcam, ab32072, UK), anti-Bcl-2 antibody (1:1000; Abcam, ab182858, UK), anti-BAX antibody (1:1000; Abcam, ab32503, UK), anti-Cleaved Caspase-3 antibody (1:500; Abcam, ab32042, UK), NQO1 Monoclonal antibody (1:5000; Proteintech, 67240-1-Ig, Wuhan, China), HSP90 Polyclonal antibody (1:5000; Proteintech, 13171-1-AP, Wuhan, China) or anti-Tubulin antibody (1:1000; Immunoway, YT4780, USA) overnight at 4 °C with rotation. Blots were then incubated in goat anti-rabbit HRP secondary antibodies (1:5000; Immunoway, RS0002, USA) for 1 h at room temperature with rotation. After being washed with TBST, the bands were imaged on a Gel intelligent image workstation (Biolight, Shenzhen, China) and quantified (Image J) with normalization to tubulin and the control per group. Data are the average of three biological replicates.

### 2.9 Animal model

BALB/c nude mice (female, 6–8 weeks old) were purchased from Beijing Vital River Laboratory Animal Technology Co., Ltd. and housed in pathogen-free conditions with standard temperature and humidity at the Laboratory Animal Resources Center of Tsinghua University conditions. All the animal experiments were approved by the Animal Ethics Committee of Tsinghua University (Approve No. 23-LJH5).

### 2.10 *In vivo* fluorescence imaging

AZO-PRO (100 μL, 1 mmol/L) was injected intravenously into tumor-bearing mice, and fluorescence imaging was observed at different times after injection. (*n* = 3) The *in vivo* fluorescence imaging was carried out using IVIS Lumina III. Ex = 660 nm, Em = 710 nm.

### 2.11 Drug release monitoring experiment

AZO-PRO (100 μL, 1 mmol/L) was injected intravenously into tumor-bearing mice, and the mice were randomly divided into two groups, each group contain three mice. Fluorescence imaging was observed at different post-injection time. When the fluorescence reached the maximum, 660 nm laser was used to irradiate the tumor site of the mice in the experimental group for 5 min, and then continue to observe the fluorescent signal changes of the two groups of mice. After 24 h, the two group mice were sacrificed. Then, tumor tissues were taken for hypoxia-inducible factor-1*α* (HIF-1*α*) immunofluorescence staining experiments.

### 2.12 *In vivo* therapy

To establish the MCF-7 tumor model, 5 × 10^6^ MCF-7 cells suspended in cold PBS were implanted subcutaneously into the right flank of BALB/c nude mice. When the tumor size reached approximately 50 mm^3^, the mice were randomly divided into 5 groups (*n* = 4) for the treatment with PBS, Laser, AZO-PRO, PRO, and AZO-PRO + Laser. Both PRO and AZO-PRO were dispersed in solution with formula (2% DMSO+20% PEG + 5% Tween 80 + 73% ddH_2_O) and intravenous injected at 10 mg/kg. After 24 h of injection, the drug was irradiated with 660 nm laser at a power density of 0.8 W/cm^2^ for 10 min. Tumor sizes were measured using a caliper and the body weight was monitored every other day. Tumor volumes were calculated according to Eq. (1):

(1) Volume=Length×Width×Width/2

After 16 days of different treatments, the mice in each group were euthanized, and the solid tumors were collected, weighed and sectioned for pathological assessments (H&E and TUNEL staining).

### 2.13 Histological studies

After 16 days of different treatments, MCF-7 tumor-bearing mice in each group were euthanized, and the tumors and major organs (heart, liver, spleen, lung, and kidney) were collected and fixed with 4% paraformaldehyde for H&E and immunohistochemical staining. The stained tissue sections were examined on slide scanning system (Pannoramic Scan, 3DHISTECH).

### 2.14 Statistical analysis

GraphPad Prism 8 software was used for the statistical analysis. Heatmap was plotted by https://www.bioinformatics.com.cn (last accessed on 20 June 2024), an online platform for data analysis and visualization. All the experimental values are represented as mean ± standard deviation (SD) with at least three individual experiments.

## 3 Results and discussion

### 3.1 Rational design and synthesis of AZO-PRO

The BET family of proteins function as epigenetic readers, facilitating gene transcription by interacting with acetylated histones, thereby promoting RNA polymerase II-mediated transcriptional elongation^39^^,^^40^. Overexpressed in various tumors, BET proteins drive abnormal downstream gene expression, closely associated with tumor initiation and progression^41^, making them potential targets for cancer therapy. To date, a variety of PROTAC molecules have been developed to target BET proteins, including ARV-771^42^, MZ1^43^, ARV-825^44^, and so forth. In this study, JQ1 was selected as the ligand for BRD4, while VHL ligand 2 was chosen to recruit the E3 ligase, with polyethylene glycol acting as a flexible linker to synthesize PRO, following the previously reported method^45^ (Supporting Information Schemes S1 and S2). As an analog of ARV-771, PRO effectively degrades BRD4. The hydroxyl group on the VHL E3 ligase ligand forms critical hydrogen bonds with HIS-115 and SER-111 in the VHL protein's binding pocket, which is essential for its binding affinity^14^^,^^46^. To disrupt this binding and deactivate PROTAC activity, a protective group was introduced at the hydroxyl site. A hypoxia-responsive group, comprising 4,4′-dihydroxymethylazobenzene and MB, was synthesized and linked to the hydroxyl group of the VHL E3 ligase ligand *via* carbonate bonds to restrict PRO activity (Supporting Information Scheme S3). Among them, the 10-N position of MB was chemically modified to obtain a leucomethylene (LMB) derivative, which exhibits a persistent electron-conjugated reduction state^47^. Only under hypoxic conditions can it be converted back to MB, thereby restoring its fluorescence signal and photosensitivity^48^, ^49^, ^50^. This property endows the prodrug with the function of real-time monitoring of drug release and reduces the phototoxicity of the prodrug. The successful synthesis of the hypoxia-responsive PROTAC prodrug AZO-PRO was confirmed by mass spectrometry, ^1^H NMR, and ^13^C NMR. (Supporting Information Figs. S1–S21).

### 3.2 The reactivity and stability of AZO-PRO

**Figure 2:**
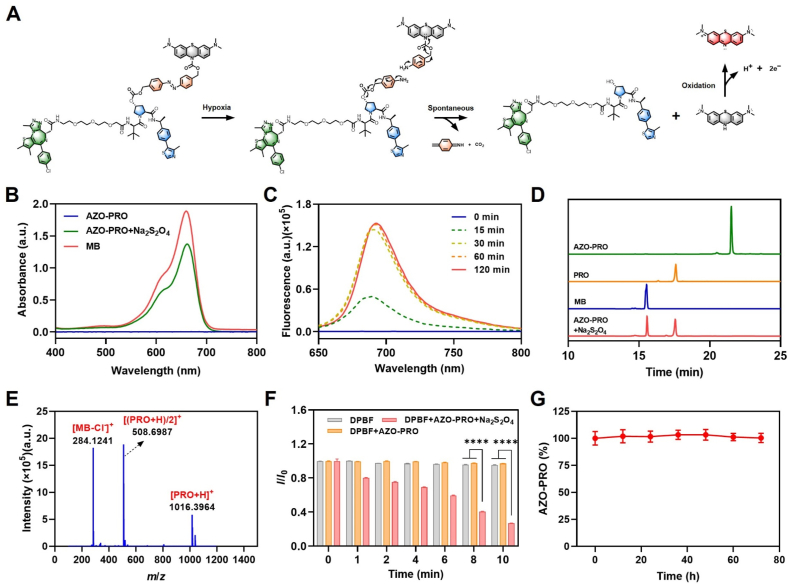
The reactivity and stability of AZO-PRO. (A) Reaction mechanism of hypoxia-triggered activation of PROTAC prodrug. (B) UV absorption spectra of MB (80 μmol/L), AZO-PRO (50 μmol/L), and the mixture of AZO-PRO (50 μmol/L) and Na_2_S_2_O_4_ (50 mmol/L) after 2 h co-incubation. (C) The fluorescence response of AZO-PRO (50 μmol/L) to Na_2_S_2_O_4_ (50 mmol/L) in PBS buffer (5 mmol/L) at 37 °C for different times. (D) The high-performance liquid chromatography (HPLC) analysis of AZO-PRO (100 μmol/L), PRO (100 μmol/L), MB (100 μmol/L), and AZO-PRO (100 μmol/L) with Na_2_S_2_O_4_ (50 mmol/L) for 2 h. The detection wavelength was 254 nm. (E) HRMS spectrum of AZO-PRO (100 μmol/L) after reaction with Na_2_S_2_O_4_ (50 mmol/L) for 2 h. (F) Normalized absorbance profile of DPBF in different treatment groups. Data are presented as mean ± SD (*n* = 3). ∗∗∗∗*P* < 0.0001. (G) The stability of AZO-PRO in serum-containing medium over time. Data are presented as mean ± SD (*n* = 3).

In the hypoxic tumor microenvironment, the reduction of the azo bond triggers electron transfer within the benzamide moiety, which induces hydrolysis of the carbamate bond, releasing both the PROTAC molecule and LMB (Fig. 2A). The non-fluorescent LMB is subsequently oxidized, yielding the MB fluorophore with NIR emission. Critically, the nitrogen atom in the phenothiazine ring of MB is restored, re-establishing its conjugated structure and thereby activating both its fluorescence signal and photosensitivity^48^. Sodium hydrosulfite (Na_2_S_2_O_4_) is a classic deoxygenating agent that can act as an electron donor to reduce azobenzene^51^. To verify the reactivity of AZO-PRO, Na_2_S_2_O_4_ was used as an azo reductase substitute to simulate the hypoxic tumor microenvironment^51^, ^52^, ^53^. The UV absorption and fluorescence spectra of AZO-PRO following activation were analyzed. The results showed a significant increase in the 660 nm absorption peak over time (Supporting Information Fig. S22), corresponding to the absorption peak of MB (Fig. 2B), indicating the release of MB. Concurrently, the fluorescence intensity of MB at its maximum emission peak (690 nm) gradually increased with time (Fig. 2C and Supporting Information Fig. S23). These changes are likely due to the cleavage of the azo bond, further corroborated by the color change of the compound before and after the reaction (Supporting Information Fig. S24). HPLC confirmed the release of PRO during AZO-PRO cleavage. Following co-incubation with Na_2_S_2_O_4_ at 37 °C, HPLC analysis revealed a decrease in the peak area of AZO-PRO (RT = 21.52 min), with the appearance of peaks corresponding to PRO (RT = 17.60 min) and MB (RT = 15.53 min) (Fig. 2D). Additionally, high-resolution mass spectrometry detected characteristic PRO peaks in the reaction mixture (Fig. 2E), confirming the successful cleavage of AZO-PRO into PRO and MB.

Once AZO-PRO is activated, the released MB absorbs light energy, transitioning from its ground state to an excited state. Some molecules emit fluorescence, enabling tumor imaging and drug tracing, while others transfer energy to oxygen, generating ROS for PDT. To verify this, 1,3-diphenylisobenzofuran (DPBF) was used to measure ^1^O_2_ production. The characteristic absorption peak of DPBF at 415 nm consistently decreased under laser irradiation in the AZO-PRO + Na_2_S_2_O_4_ co-incubation group, while the AZO-PRO-alone group showed minimal changes, indicating aminodiazotization significantly inhibits ^1^O_2_ production (Fig. 2F and Supporting Information Fig. S25). The reactivities of AZO-PRO to other analytes were also tested to verify its specificity for hypoxic conditions. The results showed that AZO-PRO exhibited a strong fluorescence response only to Na_2_S_2_O_4_, demonstrating its high selectivity (Supporting Information Fig. S26). Stability assessments by HPLC revealed that AZO-PRO remained intact after incubation in phosphate-buffered saline (PBS), fetal bovine serum (FBS), and complete DMEM cell culture medium for 72 h at room temperature (Fig. 2G, Supporting Information Figs. S27 and S28), indicating that the azobenzene modification did not compromise the stability of the PROTAC prodrug.

### 3.3 Fluorescence recovery and ROS generation of AZO-PRO in hypoxic cells

**Figure 3:**
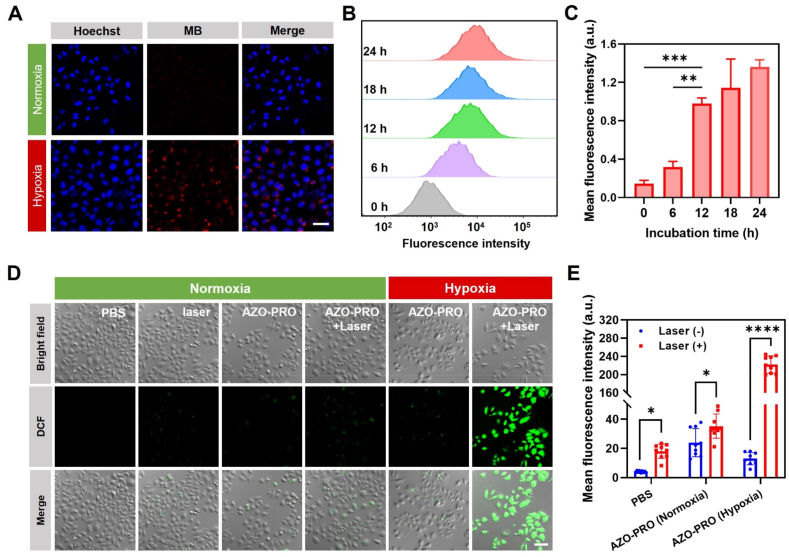
Fluorescence recovery and ROS generation of AZO-PRO in hypoxic cells. (A) Fluorescence imaging of MCF-7 cells treated with AZO-PRO (5 μmol/L) under normoxic and hypoxic conditions. Scale bar = 50 μm. (B) Representative flow cytometry data and (C) mean fluorescence intensity of MCF-7 cells treated with AZO-PRO (5 μmol/L) for 0, 6, 12, 18, and 24 h under hypoxic conditions. Data are presented as mean ± SD (*n* = 3). ∗∗*P* < 0.01, ∗∗∗*P* < 0.001. (D) Fluorescence images and (E) mean fluorescence intensity of MCF-7 cells treated with AZO-PRO (5 μmol/L) for 12 h under various conditions, followed by staining with DCFH-DA and NIR laser (660 nm, 0.8 W/cm^2^) irradiation for 5 min. DCF: Ex = 488 nm, Em = 520–600 nm. Scale bar = 100 μm. Data are presented as mean ± SD (*n* = 9). ∗*P* < 0.05, ∗∗∗∗*P* < 0.0001.

The activation of AZO-PRO in MCF-7 cells under normoxic and hypoxic conditions was evaluated using fluorescence imaging and flow cytometry (Fig. 3A and B). Under hypoxic conditions, the fluorescence intensity of the intracellular red channel progressively increased with prolonged incubation of AZO-PRO, plateauing after approximately 12 h (Fig. 3C). Based on this observation, 12 h was selected as the optimal hypoxic incubation time for subsequent cellular experiments.

Subsequently, the intracellular ROS production capacity of AZO-PRO was assessed using 2,7-dichlorofluorescein diacetate (DCFH-DA) as a fluorescent probe. Under dark conditions, DCF fluorescence was almost undetectable in the cells (Fig. 3D and E). In MCF-7 cells incubated with AZO-PRO under normoxia, only weak fluorescence signals were observed after laser irradiation, suggesting the prodrug exhibits limited ROS-generating capacity in its inactive form. Conversely, under hypoxic conditions, the AZO-PRO + Laser group emitted a pronounced green fluorescence signal, attributed to the substantial production of ROS. These results suggest that only activated AZO-PRO in hypoxic cells can generate ROS upon NIR laser irradiation, which is consistent with the expected results.

### 3.4 Anticancer properties and protein degradation activity of AZO-PRO *in vitro*

**Figure 4:**
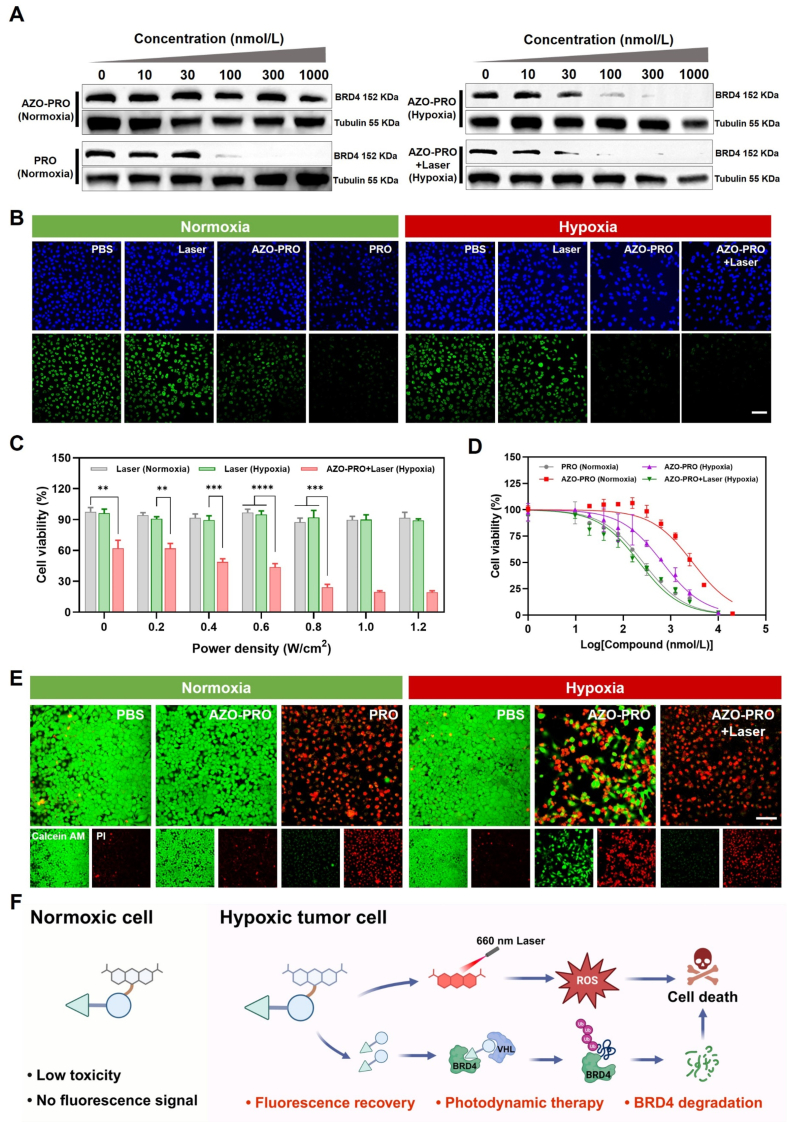
*In vitro* cytotoxicity and protein degradation activity of AZO-PRO. (A) Western blotting analysis of BRD4 expression level in MCF-7 cells treated with different concentrations of the drug under different conditions for 24 h. (B) Immunofluorescent staining of BRD4 (green) after treatment with different formulations for 24 h. Scale bar = 100 μm. DAPI: Ex = 405 nm, Em = 415–465 nm. Alexa Fluor 488: Ex = 488 nm, Em = 510–560 nm**. (**C) Cell viability of MCF-7 cells irradiated with different power densities after different treatments. Data are presented as mean ± SD (*n* = 3). ∗∗*P* < 0.01, ∗∗∗*P* < 0.001, ∗∗∗∗*P* < 0.0001. (D) Cell viability of MCF-7 cells after treatment with PRO (Normoxia), AZO-PRO (Normoxia), AZO-PRO (Hypoxia) or AZO-PRO + Laser (Hypoxia). Data are presented as mean ± SD (*n* = 3). (E) Fluorescence imaging of MCF-7 cells co-stained with Calcein AM and propidium iodide (PI) following different treatments. Scale bar = 100 μm. Calcein AM: Ex = 494 nm, Em = 505–525 nm. PI: Ex = 535 nm, Em = 600–630 nm. (F) Schematic illustration of AZO-PRO-induced BRD4 protein degradation and tumor cell death under hypoxic conditions.

After successfully verifying the activation of AZO-PRO in MCF-7 cells, we evaluated the protein degradation capacity of inactivated and active AZO-PRO. MCF-7 cells were divided into four groups, each group was treated with different concentrations of compounds under normoxic or hypoxic conditions, and the expression level of BRD4 protein was analyzed by Western blotting. Under normoxic conditions, AZO-PRO showed limited BRD4 degradation even at the highest tested concentration (1000 nmol/L) (Fig. 4A and Supporting Information Fig. S29). The activation of AZO-PRO under hypoxic conditions resulted in the recovery of protein degradation activity, thereby inducing significant degradation of BRD4 in MCF-7 cells. The results indicate that the protein degradation activity of AZO-PRO is sensitive to hypoxic conditions, and the released PRO can significantly degrade BRD4 protein. In addition, to verify the mechanism of BRD4 protein degradation, we pretreated MCF-7 cells with proteasome inhibitor MG132 or the NEDD8-activating enzyme inhibitor (NEDD8i, MLN4924)^54^ to selectively inhibit Cullin-RING ubiquitin ligase class E3 ligases. As expected, under hypoxic conditions, MG132 or MLN4924 blocked the BRD4 protein degradation activity of the AZO-PRO + Laser group (Supporting Information Fig. S30). The results showed that AZO-PRO degraded BRD4 in a hypoxia- and proteasome-dependent manner. To further validate the protein degradation activity of AZO-PRO, we treated MCF-7 cells with various conditions and evaluated intracellular BRD4 expression through immunofluorescence staining (Fig. 4B). Treatment with either laser or hypoxia alone exhibited negligible impact on BRD4 levels, suggesting that neither hypoxia nor laser treatment significantly influenced the degradation process. However, the BRD4 signal was significantly reduced in the AZO-PRO-treated group under hypoxic conditions. Furthermore, the expression of BRD4 protein diminished following irradiation with a 660 nm laser. This phenomenon may be attributed to the liberation of ROS from MB activated by NIR laser, thereby leading to the degradation of biological macromolecules such as proteins^55^. We further performed Western blotting analysis on the effect of different concentrations of MB on BRD4 protein degradation under NIR laser irradiation. The results showed that with the increase of MB concentration, the level of BRD4 protein also showed a decreasing trend, which provided more conclusive evidence for the role of PDT in target protein degradation (Supporting Information Fig. S31).

BRD4 is generally considered to be a factor that promotes cell survival and proliferation, so reduced BRD4 levels will inhibit tumor cell proliferation. Cell viability of MCF-7 cells was assessed to evaluate the cytotoxicity of AZO-PRO. Notably, the cell viability remained above 90% with increasing laser power density, irrespective of the normoxic or hypoxic conditions. In the AZO-PRO + Laser (Hypoxia) group, cell viability decreased significantly with increasing laser power density, while no further decrease in viability was observed when the power density reached 0.8 W/cm^2^ (Fig. 4C). Consequently, 0.8 W/cm^2^ was selected as the optimal parameter for 660 nm laser irradiation. To further compare the cytotoxicity of inactive and active AZO-PRO, MCF-7 cells were incubated with different doses of the compound and then subjected to different treatments. As shown in Fig. 4D, inactivated AZO-PRO exhibited the lowest cytotoxicity (IC_50_ = 2.89 μmol/L), whereas the cytotoxicity of the prodrug increased significantly (IC_50_ = 0.675 μmol/L) under hypoxic conditions. This enhancement was attributed to the low expression of azo reductase under normoxic conditions, which resulted in negligible effects of the prodrug on cell proliferation, thereby emphasizing its specific cytotoxicity against hypoxic tumor cells. Importantly, ROS generated by 660 nm laser irradiation following hypoxic treatment further enhanced the cytotoxicity of the prodrug (IC_50_ = 0.218 μmol/L), indicating that the combination therapy was more effective than PROTAC alone (IC_50_ = 0.264 μmol/L). The anticancer activity of AZO-PRO was further confirmed through double staining with calcein AM and propidium iodide (Fig. 4E). Collectively, these data indicate that AZO-PRO can be selectively activated in hypoxic tumor cells, releasing PRO and MB. PRO can induce BRD4 degradation through the proteasome pathway to trigger tumor cell death. MB can also further reduce intracellular BRD4 protein levels through the PDT process, thereby synergistically enhancing the tumor therapeutic effect of AZO-PRO (Fig. 4F).

### 3.5 Apoptosis induced by AZO-PRO under hypoxia

**Figure 5:**
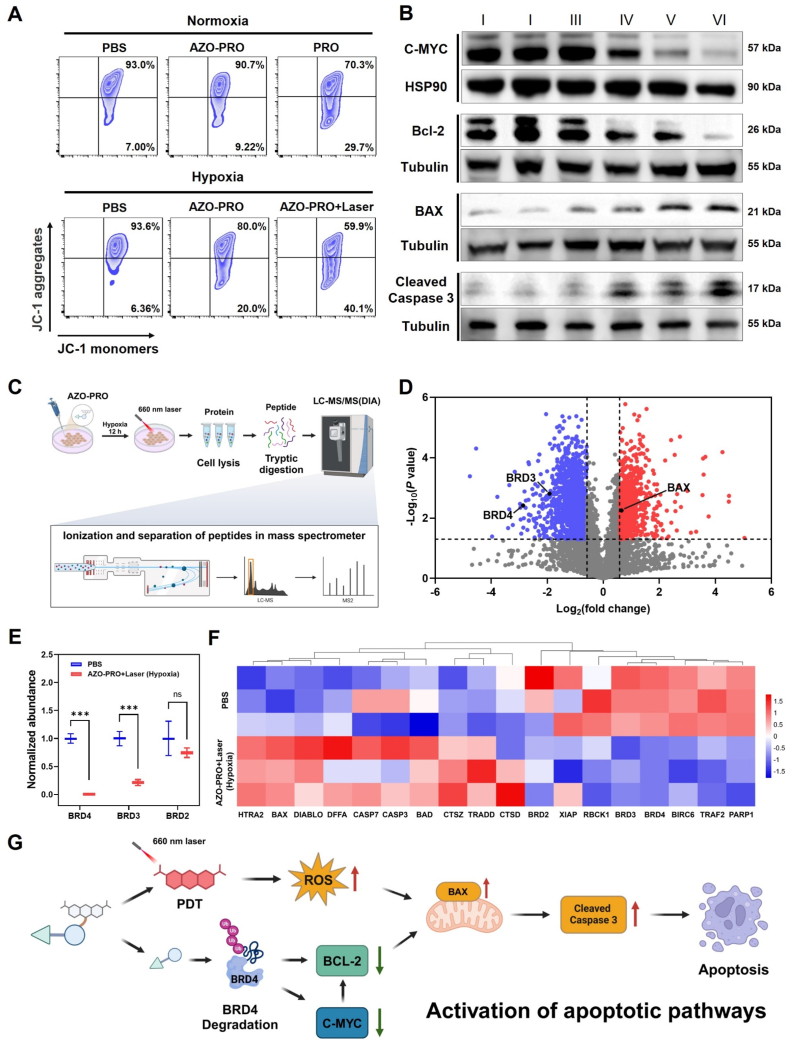
Apoptosis induced by AZO-PRO under hypoxia. (A) Flow cytometry profiles of cellular MMP changes after different treatments. (B) Western blotting detection of C-MYC, Bcl-2, BAX and Cleaved Caspase 3. I: PBS (Normoxia), II: AZO-PRO (Normoxia), III: PBS (Hypoxia), IV: AZO-PRO (Hypoxia), V: PRO (Normoxia), VI: AZO-PRO + Laser (Hypoxia). (C) Schematic illustration of MCF-7 cells incubated with AZO-PRO under hypoxic conditions for 24 h, followed by laser irradiation (660 nm, 0.8 W/cm^2^) for 5 min and subsequent treatment with cell lysates, prepared according to standard procedures for proteomic analysis. (D) Comparison of proteomic changes after cotreatment with AZO-PRO + Laser (Hypoxia) or PBS in MCF-7 cells. The dotted lines indicate either a 1.5-fold loss or increase of the protein level (*x*-axis) and *P* = 0.05 (*y*-axis). The data are presented as the mean ± SD (*n* = 3). (E) Quantification of representative BRD4, BRD3 and BRD2 proteins of mass spectrometry proteomics. ns means no significance, ∗∗∗*P* < 0.001. (F) Heat map depicting changes in protein expression of interest upon treatment with PBS or AZO-PRO + Laser (Hypoxia). (G) Schematic illustration of the synergistic induction of apoptosis through hypoxia-triggered protein degradation and PDT.

BRD4 has been shown to directly or indirectly regulate intrinsic or extrinsic apoptotic pathways, while ROS can induce intracellular oxidative stress directly and facilitate apoptosis^37^^,^^56^. Given the critical role of mitochondria in regulating cancer cell apoptosis, we utilized JC-1 to assess variations in mitochondrial membrane potential (MMP). Compared to the PBS (Normoxia) group, MCF-7 cells treated with the AZO-PRO + Laser (Hypoxia) group presented evident green fluorescence, indicating significant depolarization of the MMP (Fig. 5A and Supporting Information Fig. S32), which was consistent with the confocal laser scanning microscope images (Supporting Information Fig. S33).

Subsequently, we investigated the role of AZO-PRO in inducing apoptosis in tumor cells and its mechanism. C-Myc is an important transcription factor that can directly interact with BRD4, thereby increasing transcriptional activity and promoting rapid proliferation and anti-apoptotic function of tumor cells^57^. The relative expression levels of apoptosis-related proteins in MCF-7 cells subjected to various treatments were evaluated by Western blotting (Fig. 5B). The AZO-PRO + Laser (Hypoxia) group significantly downregulated the expression of C-Myc and anti-apoptotic protein Bcl-2, while the levels of pro-apoptotic proteins BAX and Cleaved Caspase-3 were significantly increased. These results suggest that activated AZO-PRO may induce BRD4 degradation and reduce C-Myc and Bcl-2 levels, thereby activating BAX and ultimately triggering Caspase-3-dependent cell apoptosis. Furthermore, apoptosis levels were quantified following the different treatments (Supporting Information Fig. S34). As anticipated, both PRO (Normoxia) and AZO-PRO (Hypoxia) induced substantial apoptosis in MCF-7 cells, with respective rates of late-stage apoptosis at 33.8% and 31.8%. Notably, in the AZO-PRO + Laser (Hypoxia) group, the combination of AZO-PRO with PDT significantly increased the late-stage apoptosis rate to 42.1%, highlighting a superior apoptotic-inducing capability compared to AZO-PRO alone.

To further verify the anti-tumor mechanism, we compared protein levels by labeling free quantitative mass spectrometry proteomics assay. MCF-7 cells were treated with AZO-PRO and cultured under hypoxic conditions for 12 h before irradiation with a 660 nm laser for 5 min and then cultured for another 12 h. Following this, intracellular proteins were isolated and assessed for expression levels (Fig. 5C). After quantifying 5073 proteins, we observed a significant degradation of BRD4 and BRD3 with co-treatment (Fig. 5D and E). These findings indicate that AZO-PRO has excellent hypoxia-responsiveness and can effectively induce BET protein degradation upon activation. Additionally, treatment with AZO-PRO upregulated the pro-apoptotic protein BAX (Supporting Information Fig. S35), consistent with the Western blotting results. Sixteen apoptosis-related proteins were marked from the differentially expressed proteins, which had a physical or functional relationship with BET proteins (Fig. 5F and Supporting Information Fig. S36). Collectively, these data suggest that the combination of activated AZO-PRO and PDT induces tumor cell damage through apoptotic pathway activation (Fig. 5G).

### 3.6 *In vivo* fluorescence imaging of tumor-bearing mice

**Figure 6:**
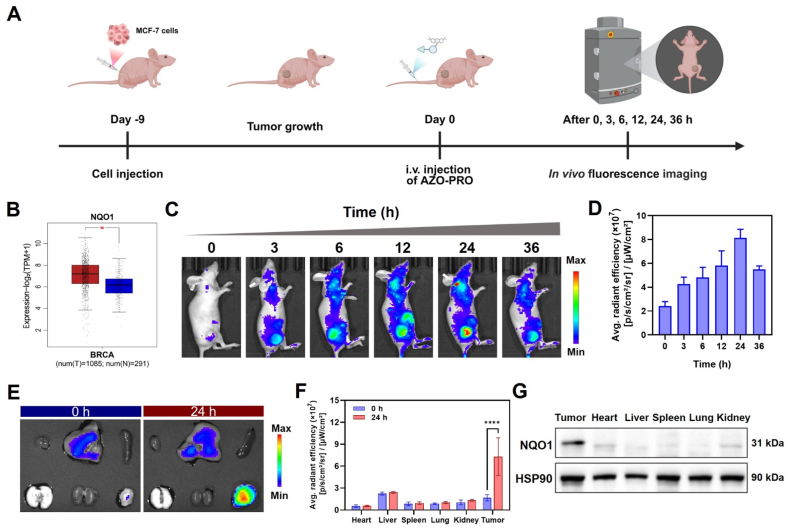
*In vivo* fluorescence imaging of tumor-bearing mice. (A) Schematic illustration of the establishment of the MCF-7 tumor model for *in vivo* biodistribution studies. (B) Box plot showing NQO1 RNA levels across biologically independent tissues from breast cancer in the GEPIA database. (C) Fluorescence imaging and (D) fluorescence intensity change of MCF-7 tumor-bearing BALB/c nude mice after intravenous injection of AZO-PRO (100 μL, 1 mmol/L). Data are presented as mean ± SD (*n* = 3). (E) Fluorescence imaging and (F) mean fluorescence intensity of major organs and tumor tissues in MCF-7 tumor-bearing BALB/c nude mice following intravenous injection of AZO-PRO. Data are presented as mean ± SD (*n* = 4). ∗∗∗∗*P* < 0.0001. G) Western blotting of NQO1 levels in mouse tumor, spleen, lung, heart, kidney and liver tissues.

Following the demonstration of the selective cytotoxicity and apoptosis-inducing mechanisms of AZO-PRO, we further investigated its activation in mouse tumor models (Fig. 6A). NADH-quinone oxidoreductase (NQO1) is a crucial protective enzyme that primarily participates in oxidative stress defense^58^. It can also serve as an azo reductase and cleave azo bonds^59^^,^^60^. According to the Gene Expression Profile Interaction Analysis (GEPIA) database^61^, the expression of NQO1 is significantly upregulated in breast cancer (Fig. 6B), which may serve to maintain redox balance in hypoxic tumors. To confirm the activation of AZO-PRO at the tumor site, we conducted fluorescence imaging. As illustrated in Fig. 6C and D, fluorescence signals at the tumor site progressively increased over time, peaking at 24 h, which corresponded to the accumulation of the prodrug and the cleavage of its azo bonds, leading to the restoration of fluorescence.

Subsequently, we assessed the biodistribution of AZO-PRO *in vivo*. As depicted in Fig. 6E and F, 24 h post-injection, the activation of AZO-PRO was primarily localized to the tumor, with minimal distribution in the heart, liver, spleen, lungs, and kidneys. Furthermore, Western blotting analysis revealed that NQO1 levels in tumors were significantly higher than those in other organs, suggesting the tumor microenvironment selectively triggers the activation of the prodrug (Fig. 6G). The tumor-specific activation of AZO-PRO could significantly enhance its bioavailability, offering substantial potential to improve therapeutic safety.

### 3.7 PDT enhances the activation of AZO-PRO

**Figure 7:**
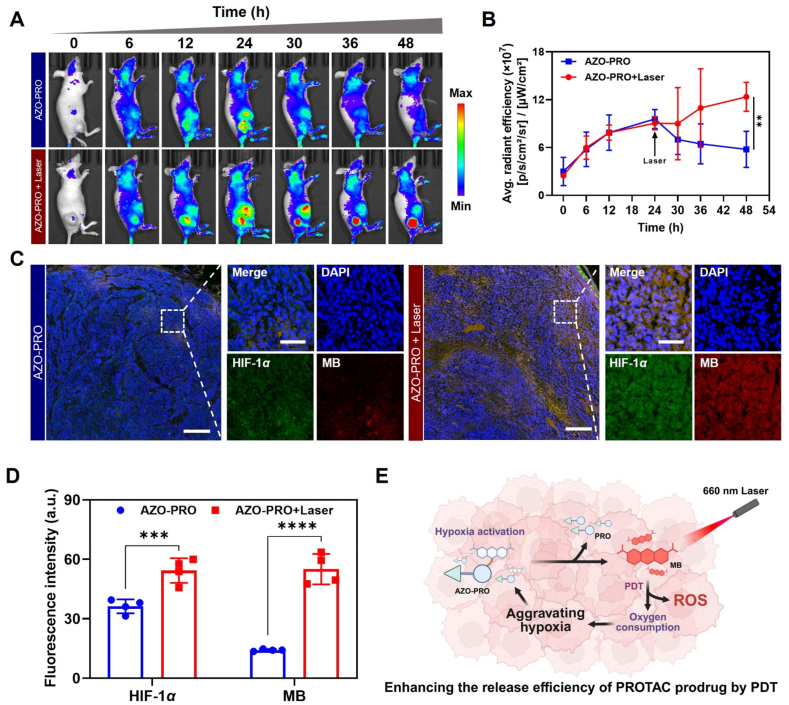
Enhancing the release efficiency of PROTAC prodrug by PDT. (A) Fluorescence imaging and (B) fluorescence intensity change of MCF-7 tumor-bearing BALB/c nude mice after tail vein injection of AZO-PRO (100 μL, 1 mmol/L) with/without laser irradiation. 660 nm laser irradiation was performed at 24 h. Data are presented as mean ± SD (*n* = 3). ∗∗*P* < 0.01. (C) *Ex-vivo* fluorescence images of tumor section post 48 h injection (left panel scale bar = 600 μm, right panel scale bar = 150 μm, the blue channel represents DAPI, the green channel represents HIF-1*α* and the red channel represents MB). (D) The mean fluorescence intensity from (C). Data are presented as mean ± SD (*n* = 3). ∗∗∗*P* < 0.001, ∗∗∗∗*P* < 0.0001. (E) Schematic illustration of PDT-enhanced release efficiency of PROTAC prodrugs.

When the photosensitizer reaches its maximum accumulation at the tumor site, laser irradiation can further exacerbate tumor hypoxia, thereby promoting the release of PROTAC. To validate the potential of PDT in enhancing PROTAC release, we conducted *in vivo* imaging experiments. Tumor-bearing mice were randomly allocated into two groups, with an equimolar amount of AZO-PRO (100 μL, 1 mmol/L) administered intravenously for subsequent real-time monitoring of tumor fluorescence signal variations. When the fluorescence signal did not increase, one group was exposed to a 660 nm laser (0.8 W/cm^2^, 5 min), while the other group remained unexposed to laser irradiation, and then changes in the fluorescence signals were monitored. As shown in Fig. 7A and B, in the non-irradiated group, the fluorescence intensity at the tumor site first increased and then began to gradually decrease after 24 h of treatment. In contrast, in the irradiated group, the initial trend was consistent with that of the non-irradiated group, but the fluorescence signal began to gradually increase at 30 h. These findings indicated NIR laser-stimulated PDT further disrupts the azo bond within unreacted AZO-PRO, leading to enhanced fluorescence signal specifically at the tumor site.

Additionally, immunofluorescence staining of HIF-1*α* also revealed an augmented hypoxic state within the tumor during PDT, facilitating azo bond cleavage and promoting the release of PROTACs. As shown in Fig. 7C and D, both the green fluorescence of HIF-1*α* and red fluorescence of MB were significantly enhanced in the laser-irradiated tumor tissue compared to the non-irradiated tumor tissue, indicating an exacerbation of tumor hypoxia during the PDT process. These findings validate that the strategy not only facilitates hypoxia-activated prodrug release *via* PDT but also real-time monitoring the release of the drug (Fig. 7E).

### 3.8 *In vivo* antitumor effect of AZO-PRO

**Figure 8:**
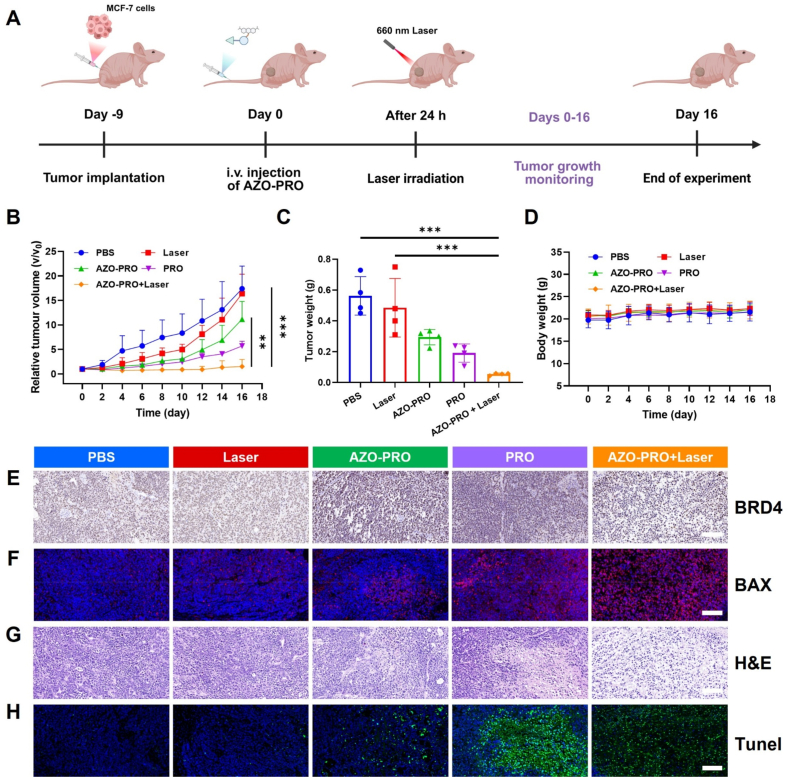
*In vivo* antitumor effect of AZO-PRO. (A) Timeline illustrating the establishment of the MCF-7 tumors mouse model and therapeutic process. (B) Relative tumor volume changes of mice with different treatments. Data are presented as mean ± SD (*n* = 4). ∗∗*P* < 0.01, ∗∗∗*P* < 0.001. (C) Tumor weight of the mice with different treatments. Data are presented as mean ± SD (*n* = 4). ∗∗∗*P* < 0.001. (D) Body weight changes in MCF-7 tumor-bearing mice with different treatments. Data are presented as mean ± SD (*n* = 4). (E) Immunohistochemical staining of BRD4 in the tumor tissue sections of each group. (F) Expression of BAX in tumor regions was evaluated by immunofluorescent staining after different treatments. (G) H&E staining and (H) TUNEL staining of the tumor tissues receiving various treatments. Scale bar = 200 μm.

Following the successful demonstration of hypoxia-mediated protein degradation and cytotoxicity by AZO-PRO *in vitro*, we extended our investigation to evaluate its antitumor potential *in vivo* using a tumor-bearing mouse model. First, the pharmacokinetic profile of AZO-PRO was evaluated in mice. After intravenous injection of 10 mg/kg, the concentration of AZO-PRO in plasma was analyzed. As shown in Supporting Information Fig. S37, the half-life of AZO-PRO was 4.905 h and the peak concentration *C*_max_ was 8749.672 μg/L (5.49 μmol/L). Subsequently, the *in vivo* anti-tumor efficacy of AZO-PRO was evaluated in the MCF-7 xenograft nude mouse model to explore its potential therapeutic value. MCF-7 cells were subcutaneously injected into the right flank of BALB/c nude mice. Different formulations of the drug were administered intravenously, and laser irradiation (0.8 W/cm^2^, 10 min) was applied 24 h post-injection (Fig. 8A). The combination of AZO-PRO and PDT exhibited a significant inhibition of tumor growth compared to PRO alone (Fig. 8B). At 16 days post-injection, the tumor volume in the AZO-PRO + Laser group increased by 1.51-fold, markedly lower than that observed in the PBS group (17.39-fold) and laser group (16.39-fold). In contrast, treatment with AZO-PRO alone exhibited a modest antitumor effect, consistent with the tumor weight evaluation (Fig. 8C, Supporting Information Figs. S38 and S39). The systemic toxicity of AZO-PRO was also assessed in a xenograft mouse model. The body weight of mice was measured every alternate day, and hematoxylin-eosin (H&E) staining analyses were conducted on the main organs. No significant weight loss was observed (Fig. 8D), and no substantial tissue damage was detected in either the AZO-PRO or AZO-PRO + Laser groups (Supporting Information Fig. S40). Hematology and serum biochemistry analyses revealed comparable biochemical parameters between AZO-PRO and PBS group (Supporting Information Fig. S41). These results underscore the favorable biosafety of AZO-PRO. The expression of BRD4 was detected by immunohistochemistry in different treatment groups (Fig. 8E). Evident decreases in BRD4 levels were observed in both the AZO-PRO and AZO-PRO + Laser groups, confirming the activation of AZO-PRO and its role in promoting BRD4 protein degradation. Additionally, immunofluorescence staining of tumor tissues revealed a significant increase in the pro-apoptotic protein BAX in the AZO-PRO + Laser group compared to the PBS and Laser groups (Fig. 8F). H&E and TUNEL staining were also performed to further assess the therapeutic effect of AZO-PRO (Fig. 8G and H). Consistent with the tumor inhibition results, the AZO-PRO + Laser group exhibited the most severe tumor damage among all groups and showed more obvious apoptotic compared to the PBS group. These results demonstrate that 660 nm laser irradiation can effectively enhance the activation of AZO-PRO and increase its protein degradation activity. Furthermore, when combined with PDT, activated AZO-PRO exhibits a dramatic inhibitory effect on tumor growth.

## 4 Conclusions

In summary, we devised a strategy to enhance the efficacy of PROTAC prodrug release *via* PDT. As a proof-of-concept, we successfully synthesized a hypoxia-responsive PROTAC prodrug (AZO-PRO), which effectively links a PROTAC molecule with NIR photosensitizer through a hypoxic-sensitive azo bond. Our results demonstrated that AZO-PRO was specifically activated in hypoxic tumor environments, simultaneously releasing both PROTAC and the photosensitizer. Moreover, the real-time monitoring of this release process has been successfully achieved through *in vivo* fluorescence imaging. Additionally, the NIR laser-mediated PDT effect consumed local oxygen, exacerbating hypoxia at the tumor site and thereby promoting the release of more PROTAC molecules and photosensitizers. Degradation of BRD4 protein and generation of ROS further triggered apoptotic pathways, synergistically enhancing apoptosis in tumor cells. Collectively, our results demonstrated that PDT-enhanced drug release exhibited superior antitumor effects compared to hypoxia-activated prodrug alone. This study offers valuable insights into the synergistic integration of multiple therapeutic strategies and presents innovative approaches for promoting hypoxia-responsive prodrugs activation.

## Author contributions

Zhongliang Fu conceived the study, designed and performed the experiments, and wrote the draft of the manuscript. Chunrong Yang designed and analyzed the experiments, edited the manuscript, and obtained funding. Yuchen Yang and Meichen Pan analyzed the data and edited the manuscript. Hongwei Hou edited the manuscript. Jinghong Li designed, supervised, and directed the project, edited the manuscript, and obtained funding. All authors have approved the final version of the manuscript.

## Conflicts of interest

The authors declare no conflicts of interest.

## Appendix A Supplementary data

The following is the Supporting Information to this article.
