# Supplementary material for: Photodynamic enhancement of PROTAC prodrug activation in hypoxic tumors
Source: Acta Pharm Sin B. 2025 Jul 14;15(9):4945–60. doi: 10.1016/j.apsb.2025.07.020 (PMC12491708; doi:10.1016/j.apsb.2025.07.020)
Supplement: Multimedia component 1 [file mmc1.docx]

**Supporting Information for**

**Original article**

Photodynamic enhancement of PROTAC prodrug activation in hypoxic tumors

**Zhongliang Fu^a,b,c,†^, Chunrong Yang^b,c,†^, Yuchen Yang^b,c^, Meichen Pan^a,b,c^, Hongwei Hou^b^, Jinghong Li^a,b,c,d,^***

^a^*School of Biomedical Sciences,* *Hunan University, Changsha 410082, China*

^b^*Beijing Life Science Academy, Beijing 102209, China*

^c^*New Cornerstone Science Laboratory, Department of Chemistry, Key Laboratory of Bioorganic Phosphorus Chemistry & Chemical Biology, Tsinghua University, Beijing 100084, China.*

^d^*Center for BioAnalytical Chemistry, Hefei National Laboratory of Physical Science at Microscale, University of Science and Technology of China, Hefei 230026, China*Received 11 November 2024; received in revised form 13 February 2025; accepted 23 May 2025

^*^Corresponding authors.

E-mail address: jhli@mail.tsinghua.edu.cn (Jinghong Li).

^†^These authors made equal contributions to this work.

1. **Supporting materials and methods**

*Materials*

The organic solvents (such as dichloromethane, petroleum ether, methanol, acetone, ethyl acetate, tetrahydrofuran, etc.), inorganic salts (such as Na_2_CO_3_, NaCl, Na_2_S_2_O_4_, Na_2_SO_4_, etc.) and column chromatography silica gel used in this project were purchased from Shanghai Titan Scientific Co., Ltd. All organic solvents were analytically pure and received without further treatment. Deuterium reagent (CDCl_3_, DMSO-*d*_6_), trifluoroacetic acid (CAS: 76-05-1), (*S*)-*tert*-butyl 2-(4-(4-chlorophenyl)-2,3,9-triMethyl-6H-thieno[3,2-*f*][1,2,4]triazolo[4,3-*a*][1,4]diazepin-6-yl)acetate (CAS: 1268524-70-4), (2*S*,4*R*)-1-((*S*)-2-amino-3,3-dimethylbutanoyl)-4-hydroxy-*N*-((*S*)-1-(4-(4-*m*ethylthiazol-5-yl)phenyl)ethyl)pyrrolidine-2-carboxamide (CAS：1948273-02-6), 1-[bis(dimethylamino)methylene]-1*H*-1,2,3-triazolo[4,5-*b*]pyridinium 3-oxide hexafluorophosphate (abbreviated: HATU; CAS: 148893-10-1), *N*,*N*-diisopropylethylamine (abbreviated: DIPEA; CAS: 7087-68-5), ultra-dry *N*,*N*-dimethylformamide (DMF, CAS: 68-12-2), Triphosgene (CAS: 32315-10-9), ultra-dry dichloromethane (DCM, CAS: 75-09-2), 4-dimethylaminopyridine (abbreviated: DMAP; CAS: 1122-58-3), and triethylamine (CAS: 121-44-8) were purchased from Anhui Zesheng Technology Co., Ltd. Methylene blue (CAS: 61-73-4), (diazene-1,2-diylbis(4,1-phenylene))dimethanol (CAS: 37797-30-1), and 2,2-dimethyl-4-oxo-3,8,11,14-tetraoxa-5-azahexadecan-16-oic acid (CAS: 462100-06-7) were purchased from Bide Pharmatech Co., Ltd. 4-Nitrophenyl chloroformate (CAS: 7693-46-1) was purchased from TCI (Shanghai, China) Development Co., Ltd. Tween 80 was purchased from MedChemExpress LLC. Fetal bovine serum (Gibco, Cat. No. 10437010, USA) and DMEM culture-medium (Gibco, Cat. No. 11995065, USA) were purchased from Thermo Fisher Scientific (USA) Co., Ltd. The protein marker (Cat. No. 20351ES76), SDS-PAGE protein loading buffer (Cat. No. 20315ES20), 0.25% trypsin (Cat. No. 40126ES60), and Penicillin–Streptomycin Solution (100 ×) (Cat. No. 60162ES76) were purchased from Yeasen Biotechnology (Shanghai, China) Co., Ltd. CellTiter 96® kit (Cat. No. G3581) was purchased from Promega Biotech Co., Ltd. (USA). Annexin V-FITC/PI Apoptosis Detection Kit (Cat. No. CA1020) and cell culture grade DMSO (Cat. No. D8371) were purchased from Solarbio Science & Technology Co., Ltd. (Beijing, China). RIPA lysate (Cat. No. P0013B), Reactive Oxygen Species Test Kit (Cat. No. S0033S), Immunostaining permeable Solution Triton X-100 (Cat. No. P0096), 4% paraformaldehyde fixative Solution (Cat. No. P0099), QuickBlock™ Sealing Solution (Cat. No. P0226), ECL chemiluminescence kit (Cat. No. P0018FM), Calcein/PI Cell Viability/Cytotoxicity Assay Kit (Cat. No. C2015M), and mitochondrial membrane potential assay kit with JC-1(Cat. No. C2006) were purchased from Beyotime Biotech Inc. (Shanghai, China). PBS solution (pH 7.2) was purchased from Sangon Biotech Co., Ltd. (Shanghai, China). GenScript SurePAGE, Bis-Tris, 10 × 8 cm gels were purchased from GenScript Biotech Corporation (USA). The PVDF membrane (Cat. No. IPVH00010) was purchased from Merck Millipore (Germany). BCA protein concentration determination kit (Cat. No. P0012) was purchased from Epizyme Biomedical Technology Co., Ltd. (USA). DAPI-Fluoromount-G fluorescent sealers (Cat. No. 0100-20) was purchased from SouthernBiotech (USA). Cell Crawl tablets are purchased from NEST (Cat. No. 801007, USA). PEG-300 (Cat. No. T7022) and protease inhibitor Cocktail (Cat. No. C0001) were purchased from TargetMol Chemicals Inc. (USA).

*Material characterization*

Intelligent temperature control digital display magnetic stirrer (MS-H-Pro^+^, DLAB Scientific), rotary evaporator (RE-2000, Yarong, Shanghai, China), magnetic stirrer (85-2A, Zhengzhou, China), electronic analytical balance (BS124S, Sartorius), low temperature coolant circulation pump (LTC-4/15, Xingde Jingyi, Beijing, China), low temperature constant temperature stirring reaction bath (DHJK-4005, Zhengzhou Ketech, China), 400M liquid nuclear magnetic resonance Spectrometer (JNM-ECZ400S, JEOL), ultra-high performance liquid Phase Tandem Mass Spectrometer (Waters ACQUITY, Waters), high performance Liquid chromatography (1260 INFINITY II, Agilent), Universal microplate reader (EnVision, Perkin), flow cytometry (CytoFLEX LX, Bechmann Coulter), super resolution confocal microscopy (TCS SP8 STED, Leica), eBlot Fast Wet-rotating instrument (GenScript), gel intelligent image workstation (GelView 6000Plus, Guangzhou Biolight, China), small animal imaging instrument (Lumina III, PE), slide scanning system (Pannoramic Scan, 3DHISTECH). All original uncropped Western blotting images have been provided in the Supporting Information Figs. S42-S44.

*Methods*

**Scheme S1** Structure and synthesis of compound **9**. Reagents and conditions: a) CF_3_COOH, DCM, rt, 12 h.

**Scheme S2** Structure and synthesis of compound **PRO**. Reagents and conditions: b) HATU, DIPEA, DMF, rt, 24 h; c) 1) CF_3_COOH, DCM, rt, 6 h; 2) Compound **9**, HATU, DIPEA, DMF, rt, 24 h.

**Scheme S3** Structure and synthesis of compound **AZO-PRO**. Reagents and conditions: d) Sodium dithionite, Triphosgene, DCM, 0 ℃, 1 h; e) 4,4′-*bis*(hydroxymethyl)azobenzene, DCM, rt, 6 h; f) 4-nitrophenyl chloroformate, DMAP, DCM, rt, 4 h; g) PRO, DMAP, TEA, DCM, rt, 12 h.

*Synthesis of compound* ***9***

(*S*)-*tert*-Butyl 2-(4-(4-chlorophenyl)-2,3,9-trimethyl-6*H*-thieno[3,2-*f*][1,2,4]triazolo[4,3-*a*][1,4]diazepin-6-yl)acetate (1.0 g, 2.2 mmol) dissolved in dichloromethane (20 mL), trifluoroacetic acid (3 mL) was slowly added to the solution. The mixture was stirred at room temperature for 12 h and the reaction was monitored by Thin-Layer Chromatography (TLC). After the reaction was completed, the solvent was evaporated under vacuum and purified by silica gel column chromatography (DCM: MeOH = 100:6) to obtain compound **9** as white solid. ^1^H NMR (400 MHz, DMSO-D6) *δ* 9.31 (s, 1H), 7.50-7.48 (d, *J* = 8 Hz, 2H), 7.44-7.42 (d, *J* = 8 Hz, 2H), 4.48-4.44 (t, *J* = 8 Hz, 1H), 3.45-3.39 (dd, *J* = 16, 8 Hz, 1H), 3.34-3.28 (dd, *J* = 16, 8 Hz, 1H), 2.61 (s, 3H), 2.41 (s, 3H), 1.62 (s, 3H). ^13^C NMR (101 MHz, DMSO-D6) *δ* 172.06, 163.34, 158.59, 158.22, 154.86, 150.15, 136.61, 135.41, 132.17, 131.03, 130.26, 129.98, 129.67, 128.58, 117.01, 114.12, 53.57, 36.52, 14.12, 12.74, 11.33. HRMS (ESI) *m*/*z* calcd. for C_19_H_18_ClN_4_O_2_^+^ [(M+H)^+^]: 401.0834, found: 401.0857.

*Synthesis of compound* **3**

HATU (2.47 g, 6.6 mmol), DIPEA (1.13 mL, 6.6 mmol) and 2,2-dimethyl-4-oxo-3,8,11,14-tetraoxa-5-azahexadecan-16-oic acid (1.0 g, 3.25 mmol) were dissolved into DMF (12 mL). (2*S*, 4*R*)-1-(*S*)-(2-Amino-3,3-dimethylbutanoyl)-4-hydroxy-*N*-(*S*)-(1-(4-(4-methylthiazol-5-yl) phenyl) baton rouge) pyrrolidine-2-carb oxamide (1.47 g, 3.3 mmol) was slowly added to the solution. The mixture was then stirred at room temperature for 24 h. After the reaction was completed, the mixture was diluted with a small amount of water, then extracted with ethyl acetate (100 mL × 3), followed by washing with water (50 mL) and saturated sodium chloride solution (50 mL). The organic layer was dried with anhydrous sodium sulfate, filtered, concentrated under reduced pressure and purified by silica gel column chromatography (DCM: MeOH = 20:1) to obtain the compound **3** as oily liquid. ^1^H NMR (400 MHz, DMSO-*d*_6_) *δ* 8.97 (s, 1H), 8.43-8.41 (d, *J* = 8 Hz, 1H), 7.44-7.42 (d, *J* = 8 Hz, 2H), 7.40-7.35 (m, 2H), 7.35 (s, 1H), 6.76-6.73 (m, 1H), 5.12-5.11 (d, *J* = 4 Hz, 1H), 4.93-4.86 (m, 1H), 4.55-4.52 (d, *J* = 12 Hz, 1H), 4.45-4.41 (t, *J* = 8 Hz, 1H), 4.27 (s, 1H), 3.95 (s, 2H), 3.62-3.50 (m, 10H), 3.38-3.35 (t, *J* = 6 Hz, 2H), 3.08-3.03 (m, 2H), 2.44 (s, 3H), 2.06-2.01 (m, 1H), 1.79-1.73 (m, 1H), 1.36 (s, 12H), 0.93 (s, 9H). ^13^C NMR (101 MHz, DMSO) *δ* 170.51, 169.03, 168.59, 155.63, 151.54, 151.16, 147.78, 144.76, 128.87, 126.37, 120.78, 77.63, 70.49, 69.84, 69.64, 69.21, 68.81, 58.60, 56.57, 55.73, 54.94, 53.57, 47.80, 37.76, 35.79, 28.26, 26.26, 22.52, 18.08, 16.74, 16.02, 12.49. HRMS (ESI) *m*/*z* calcd for C_36_H_55_N_5_O_9_S^+^ [(M+H)^+^]: 734.3794, found: 734.3760.

*Synthesis of compound* ***PRO***

Compound **3** (1.0 g, 1.4 mmol) was gradually introduced into a mixture of dichloromethane (15 mL) and trifluoroacetic acid (3 mL), followed by stirring the mixture at room temperature for 6 h. Subsequently, the solvent in the mixture was evaporated under vacuum conditions. The aforementioned products, compound **9** (561.23 mg, 2.1 mmol), HATU (798.50 mg, 2.1 mmol) and DIPEA (365.80 μL, 2.1 mmol) were slowly added to DMF solution (20 mL). The resulting mixture was stirred at room temperature for 24 h. Upon completion of the reaction, a small amount of water was employed to dilute the reaction mixture. Subsequently, the diluted mixture was subjected to extraction with ethyl acetate (50 mL × 3). The resulting organic phase was then washed successively with water (100 mL) and saturated sodium chloride solution (50 mL × 3). The resulting organic layer was washed, dried with anhydrous sodium sulfate, filtered to remove the solids, then reduced pressure to concentrate to remove the solvent, and finally purified by silica gel column chromatography (DCM: MeOH = 15:1), yielding compound PRO as white solid. ^1^H NMR (400 MHz, DMSO-*d*_6_) *δ* 8.97 (s, 1H), 8.43-8.42 (d, *J* = 4 Hz, 1H), 8.29-8.26 (t, *J* = 6 Hz, 1H), 7.48-7.45 (m, 2H), 7.42 (s, 2H), 7.41-7.38 (m, 2H), 7.36-7.34 (d, *J* = 8 Hz, 2H), 5.13-5.12 (d, *J* = 4 Hz, 1H), 4.93-4.86 (m , 1H), 4.55-4.42 (m, 3H), 4.27 (s, 1H), 3.96 (s, 2H), 3.63-3.58 (m, 4H), 3.46-3.44 (t, *J* = 4 Hz, 2H), 3.32 (s, 10H), 2.58 (s, 3H), 2.44 (s, 3H), 2.39 (s, 3H), 2.06-2.01 (m, 1H), 1.79-1.72 (m , 1H), 1.61 (s, 3H), 1.36-1.34 (d, J = 8 Hz, 3H), 0.93 (s, 9H). ^13^C NMR (101 MHz, DMSO) *δ* 170.53, 169.60, 169.07, 168.63, 163.45, 159.02, 158.64, 158.26, 157.88, 155.03, 151.77, 150.28, 147.51, 144.89, 136.48, 135.51, 132.13, 131.35, 131.24, 130.36, 130.03, 129.76, 129.60, 128.90, 128.55, 126.40, 116.68, 113.80, 70.50, 69.88, 69.68, 69.29, 68.84, 58.63, 56.61, 55.77, 53.68, 47.83, 37.79, 37.28, 35.82, 26.28, 22.54, 15.91, 14.11, 12.75, 11.33. HRMS (ESI) *m*/*z* calcd for C_50_H_63_ClN_9_O_8_S_2_^+^ [(M+H)^+^]: 1016.3925, found: 1016.3833.

*Synthesis of compound* ***5***

Methylene blue (640 mg, 2 mmol) was dissolved in water (50 mL) under an argon atmosphere. Subsequently, methylene chloride (25 mL) and sodium carbonate (847.92 mg, 8 mmol) were added to the solution, which was then stirred at 40 ℃ for a duration of 5 min. Sodium hydrosulfite (1392.88 mg, 8 mmol) was dissolved in water (70 mL) and added directly to the solution with a syringe. The reactants were then stirred until the solution turns yellow (usually within 15–30 min), and the mixture was cooled with an ice water bath. Triphosgene (356.10 mg, 1.2 mmol) was dissolved in dichloromethane (20 mL) and gradually added dropwise to the reaction mixture, followed by continuous stirring for 1 h. Under stirring conditions, the solution was slowly poured into ice water (200 mL) and the mixture was extracted with methylene chloride (100 mL × 3). The resulting mixture was washed in saturated salt water and dried with anhydrous sodium sulfate. After filtration, depressurization concentration and purification through silica gel column chromatography (petroleum ether: ethyl acetate = 10:1), compound **5** was obtained as white solid. ^1^H NMR (400 MHz, CDCl_3_) *δ* 7.38-7.36(d, *J* = 8 Hz, 2H), 6.68-6.67 (d, *J* = 4 Hz, 2H), 6.62-6.59 (dd, *J* = 8, 4 Hz, 2H), 2.94 (s, 12H). ^13^C NMR (101 MHz, CDCl3) *δ* 149.96, 149.33, 127.99, 110.82, 110.35, 40.69. HRMS (ESI) *m*/*z* calcd for C_17_H_19_ClN_3_OS^+^ [(M+H)^+^]: 348.0932, found: 348.0922.

*Synthesis of compound* ***6***

The (diazene-1,2-diylbis(4,1-phenylene))dimethanol (72.68 mg, 0.3 mmol), sodium carbonate (31.80 mg, 0.3 mmol), and 4-dimethylaminopyridine (36.65 mg, 0.3 mmol) were dissolved in tetrahydrofuran (5 mL). Subsequently, compound **5** (38.26 mg, 0.11 mmol) was dissolved in anhydrous dichloromethane (3 mL) and added dropwise to the reaction mixture with continuous stirring at room temperature for a duration of 6 h. After reaction finished, the resulting mixture was filtered and concentrated under reduced pressure to remove the solvent, and purified by silica gel column chromatography (dichloromethane: ethyl acetate = 10:1) to obtain compound **6** as a yellow-green solid. ^1^H NMR (400 MHz, DMSO-*d*_6_) *δ* 8.13 – 8.00 (m, 2H), 7.94-7.85 (m, 4H), 7.54-7.52 (d, *J* = 8 Hz, 4H), 7.36-7.34 (d, *J* = 8 Hz, 2H), 6.69-6.68 (m, 2H), 5.38-5.36 (t, *J* = 4 Hz, 1H), 5.26 (s, 1H), 4.61-4.59 (d, *J* = 8 Hz, 2H), 4.24-4.22 (dd, *J* = 8, 4 Hz, 1H), 2.88 (s, 12H). ^13^C NMR (101 MHz, DMSO) *δ* 153.56, 151.46, 150.84, 148.68, 146.60, 139.76, 132.05, 130.86, 129.52, 128.35, 127.17, 122.60, 122.52, 110.91, 109.75, 66.60, 62.47, 29.93, 28.40, 23.42, 22.44, 13.95, 10.94. HRMS (ESI) *m*/*z* calcd for C_31_H_32_N_5_O_3_S^+^ [(M+H)^+^]: 554.2221, found: 554.2230.

*Synthesis of compound* ***7***

The compounds **6** (276.84 mg, 0.5 mmol) and 4-dimethylaminopyridine (97.73 mg, 0.8 mmol) were dissolved in anhydrous dichloromethane (15 mL) and stirred in an argon atmosphere for 10 min. Then the 4-nitrophenyl chloroformate (161.25 mg, 0.8 mmol) was dissolved in anhydrous dichloromethane (5 mL) and slowly added to the reaction mixture, stirring at room temperature for 4 h. The resulting mixture was concentrated under reduced pressure to remove the solvent and purified by column chromatography (dichloromethane: ethyl acetate = 50:1) to compound **7** as orange-yellow solid. ^1^H NMR (400 MHz, DMSO-*d*_6_) *δ* 8.97 (s, 1H), 8.50-8.48 (d, *J* = 8 Hz, 1H), 8.31-8.25 (m, 3H), 7.64-7.61 (m, 2H), 7.50-7.44 (m, 4H), 7.42-7.38 (m, 4H), 7.36 (s, 1H), 5.27 (s, 1H), 4.95-4.88 (m, 1H), 4.54-4.47 (m, 2H), 4.43-4.40 (d, *J* = 12 Hz, 1H), 4.29-4.26 (d, *J* = 12 Hz, 1H), 3.98 (s, 2H), 3.81-3.78 (dd, *J* = 12, 4 Hz, 1H), 3.60-3.54 (m, 8H), 3.45-3.42 (t, *J* = 6 Hz, 2H), 3.28-3.17 (m, 4H), 2.58 (s, 3H), 2.44 (s, 4H), 2.39 (s, 3H), 2.10-2.03 (m, 1H), 1.60 (s, 3H), 1.38-1.36 (d, *J* = 8 Hz, 3H), 0.97 (s, 9H). ^13^C NMR (101 MHz, DMSO) *δ* 155.39, 153.64, 152.08, 151.93, 151.49, 148.77, 145.34, 140.28, 138.25, 132.15, 129.44, 128.44, 127.56, 127.16, 126.35, 125.57, 125.51, 122.89, 122.76, 120.20, 115.96, 110.93, 109.84, 69.81, 66.66, 59.90, 55.04, 20.89, 14.21. HRMS (ESI) *m*/*z* calcd. for C_38_H_35_N_6_O_7_S^+^ [(M+H)^+^]: 719.2283, found: 719.2290.

*Synthesis of compound* ***AZO-PRO***

PRO (50.83 mg, 0.05 mmol), compound **7** (35.91 mg, 0.05 mmol) and 4-dimethylaminopyridine (7.33 mg, 0.06 mmol) were dissolved in anhydrous dichloromethane (10 mL). Triethylamine (10.42 μL, 0.075 mmol) was subsequently added with agitation and the reaction proceeded at a temperature of 40 ℃ for a duration of 12 h. After the reaction, the solvent was removed by vacuum evaporation and the mixture was purified by column chromatography (dichloromethane: methanol = 20: 1) to obtain compound AZO-PRO as a deep yellow solid. ^1^H NMR (400 MHz, CDCl_3_) *δ* 9.78 (s, 3H), 9.06 (s, 1H), 7.91 (s, 2H), 7.89 (s, 2H), 7.72-7.70 (d, *J* = 7.6 Hz, 1H), 7.52 (s, 1H), 7.50 (d, *J* = 2.1 Hz, 2H), 7.48 (s, 2H), 7.46 (s, 1H), 7.40 (d, *J* = 2 Hz, 1H), 7.38 (s, 2H), 7.37 (s, 1H), 7.33 (s, 2H), 7.31 (s, 1H), 7.07 (d, *J* = 2.6 Hz, 2H), 7.04-7.01 (dd, *J* = 8.8, 2.7 Hz, 2H), 5.31 (s, 2H), 5.28 – 5.16 (m, 2H), 5.09-5.05 (t, *J* = 7.1 Hz, 1H), 4.85-4.82 (t, *J* = 7.6 Hz, 1H), 4.72-4.69 (t, *J* = 6.9 Hz, 1H), 4.61-4.59 (d, *J* = 9.1 Hz, 1H), 4.30-4.27 (d, *J* = 11.9 Hz, 1H), 4.21 – 3.96 (m, 1H), 3.88-3.84 (dd, *J* = 11.9, 4.4 Hz, 1H), 3.77 – 3.59 (m, 10H), 3.48 (h, *J* = 8.1, 7.5 Hz, 4H), 3.05 (s, 12H), 2.72 (s, 2H), 2.69 (s, 6H), 2.55 (s, 3H), 2.40 (s, 3H), 2.00 (s, 1H), 1.67 (s, 3H), 1.46-1.45 (d, J = 7.0 Hz, 3H), 1.05 (s, 9H). ^13^C NMR (101 MHz, CDCl_3_) *δ* 171.13, 170.77, 169.86, 164.95, 160.96, 160.57, 154.32, 153.39, 152.73, 152.52, 152.41, 144.84, 144.25, 138.62, 137.98, 137.69, 135.48, 133.71, 132.77, 131.56, 131.22, 130.18, 129.55, 129.13, 128.99, 128.75, 128.16, 126.96, 123.23, 116.41, 116.25, 70.90, 70.54, 70.28, 69.69, 69.40, 68.01, 58.65, 57.05, 53.86, 49.17, 44.29, 39.92, 37.70, 35.52, 33.25, 26.46, 22.11, 14.45, 14.14, 13.25, 11.33. HRMS (ESI) *m*/*z* calcd. for C_82_H_92_ClN_14_O_12_S_3_^+^ [(M+H)^+^]: 1595.5865, found: 1595.5787.

**2. Supporting figures**

**Figure S1** HRMS spectrum of compound **9**.


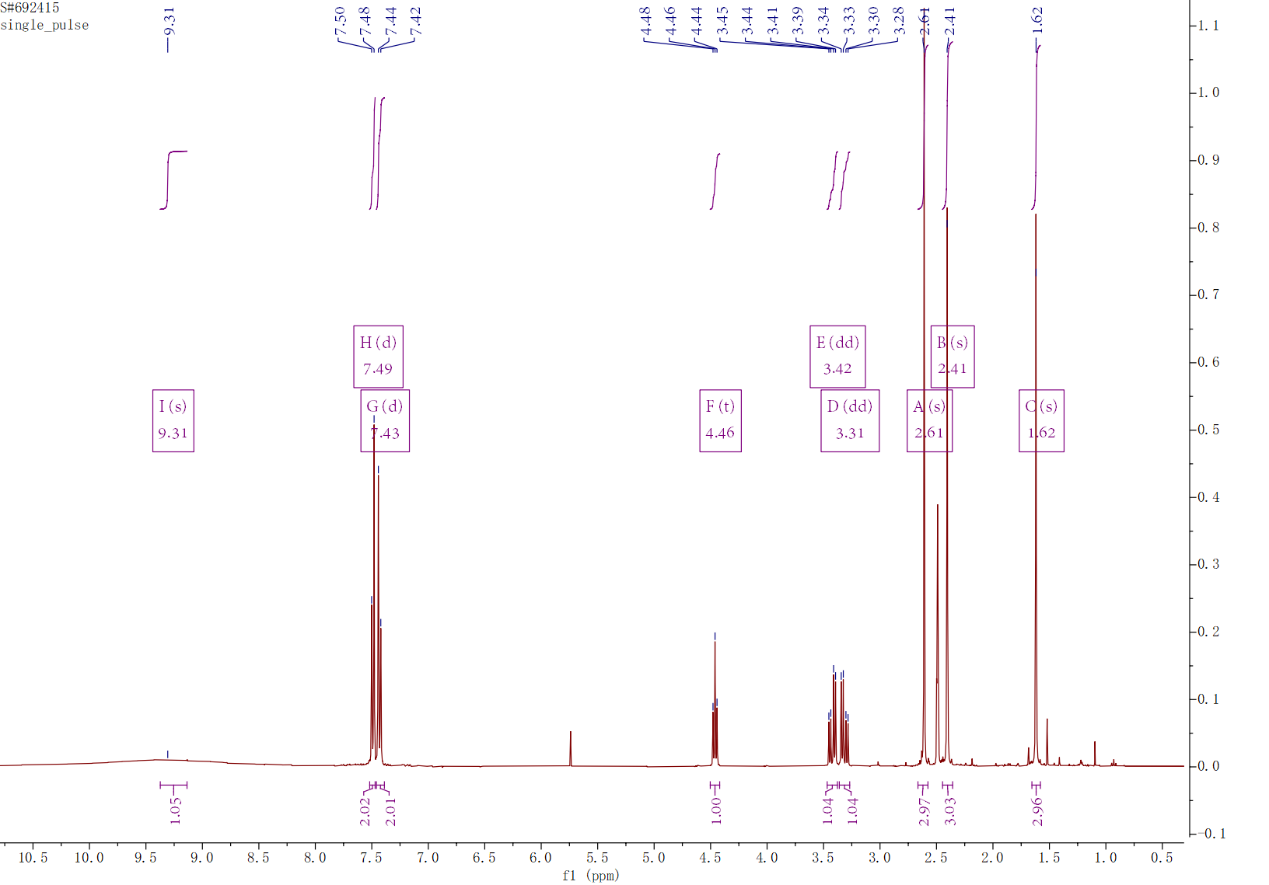


**Figure S2** ^1^H NMR spectrum of compound **9**.


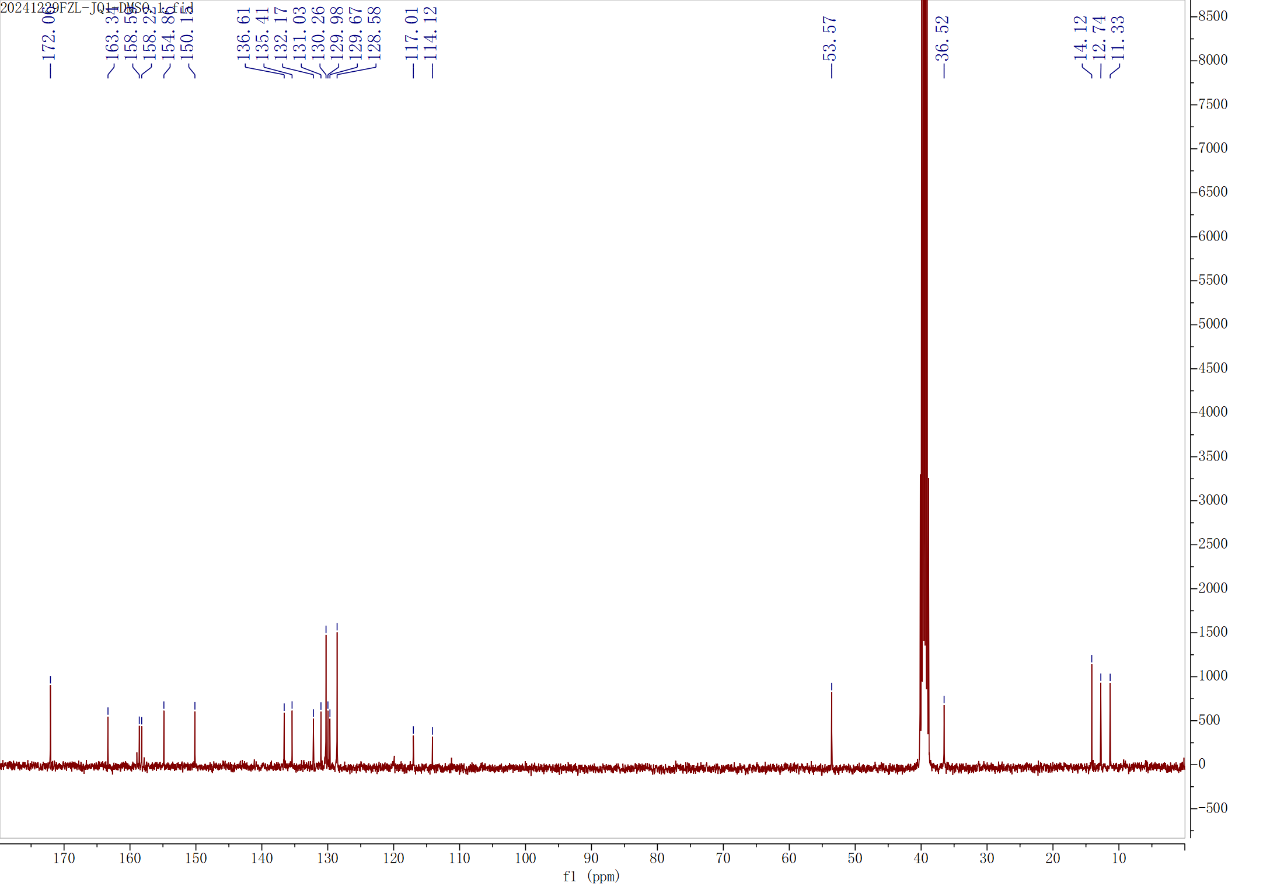


**Figure S3** ^13^C NMR spectrum of compound **9**.

**Figure S4** HRMS spectrum of compound **3**.


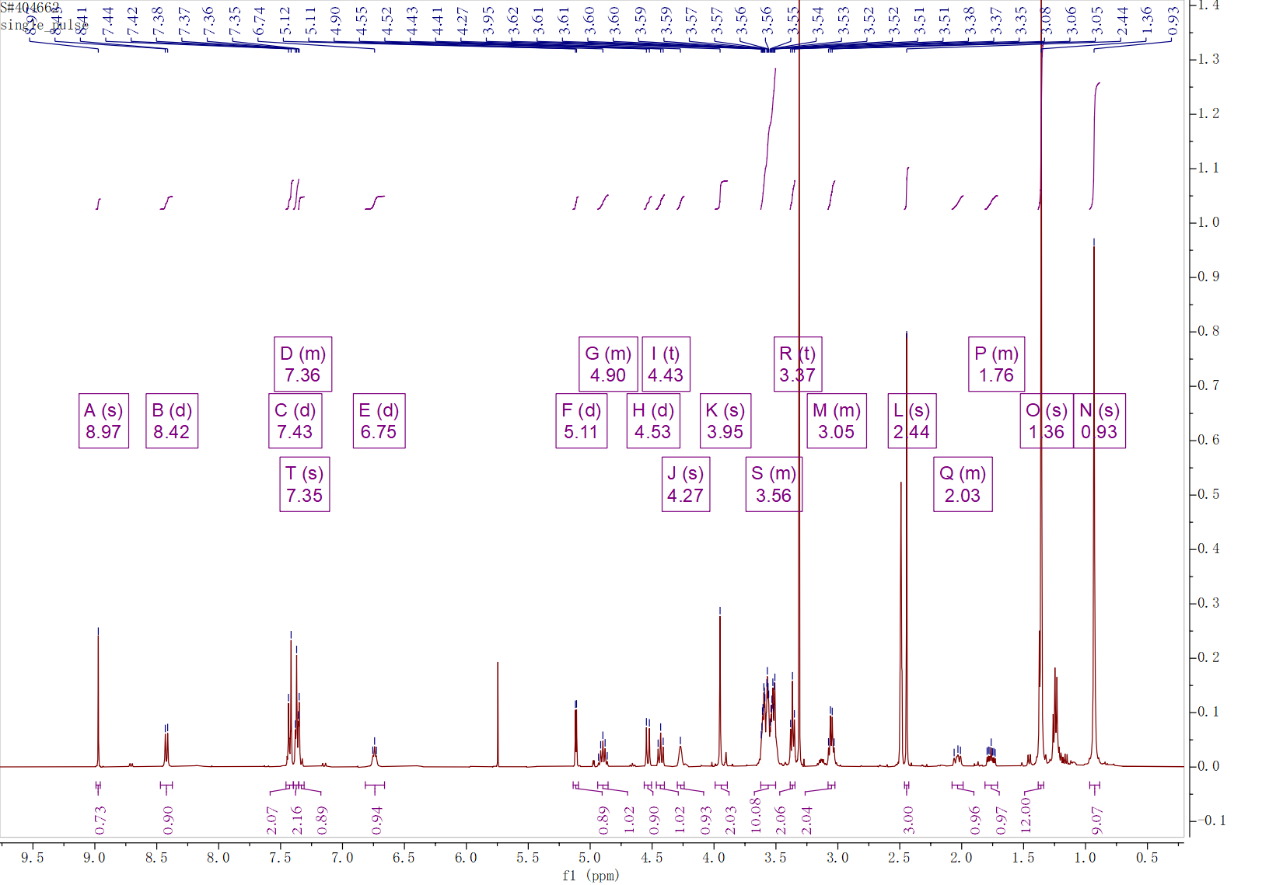


**Figure S5** ^1^H NMR spectrum of compound **3**.


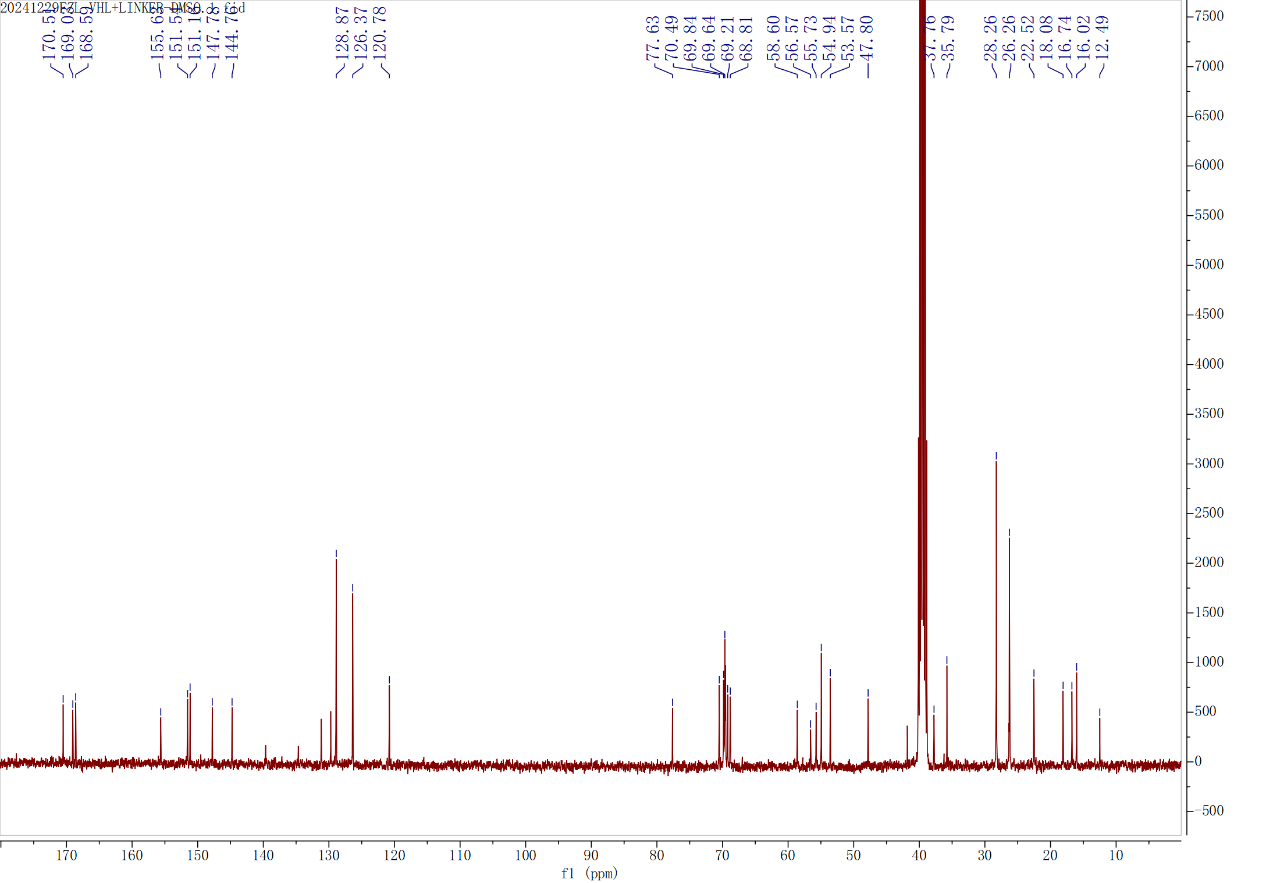


**Figure S6** ^13^C NMR spectrum of compound **3**.

**Figure S7** HRMS spectrum of PRO.


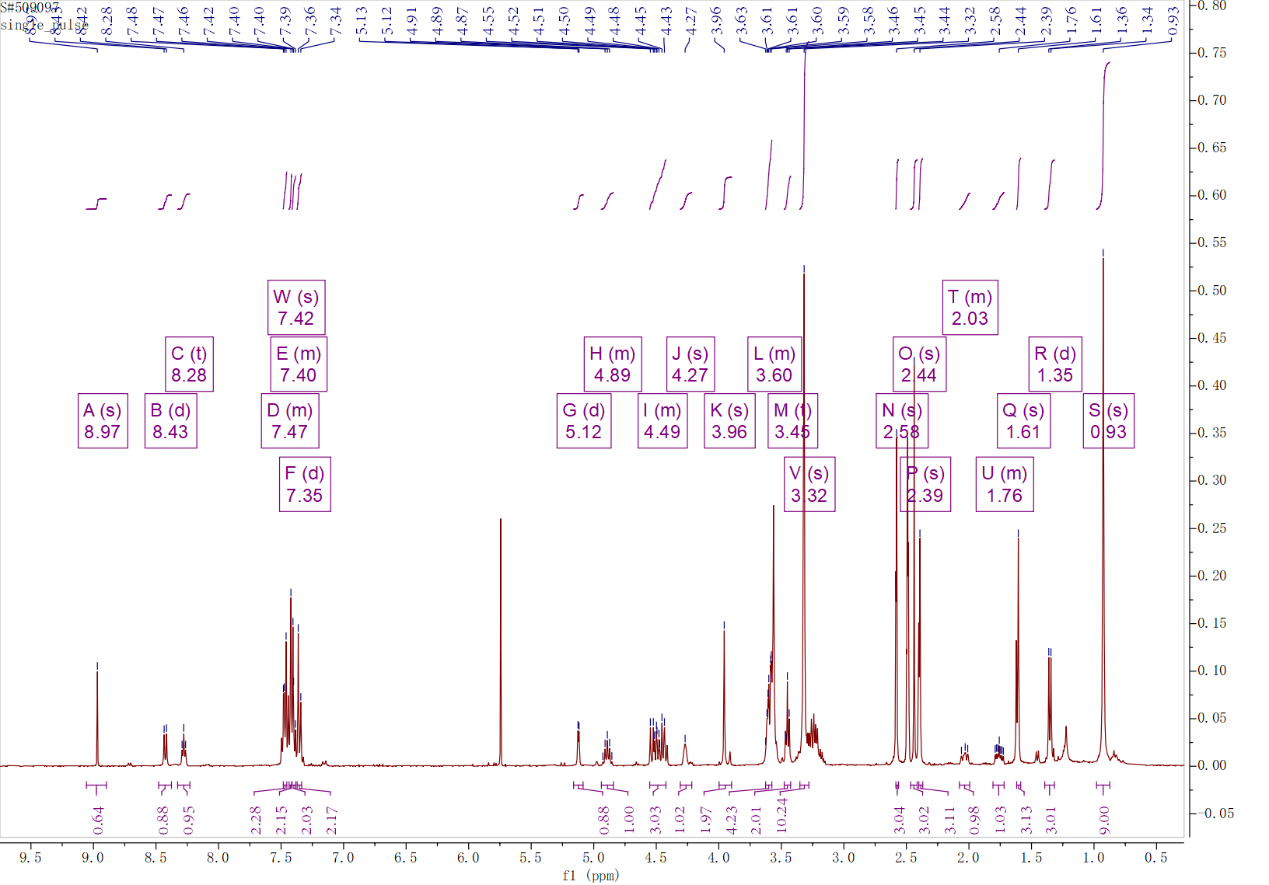


**Figure S8** ^1^H NMR spectrum of PRO.


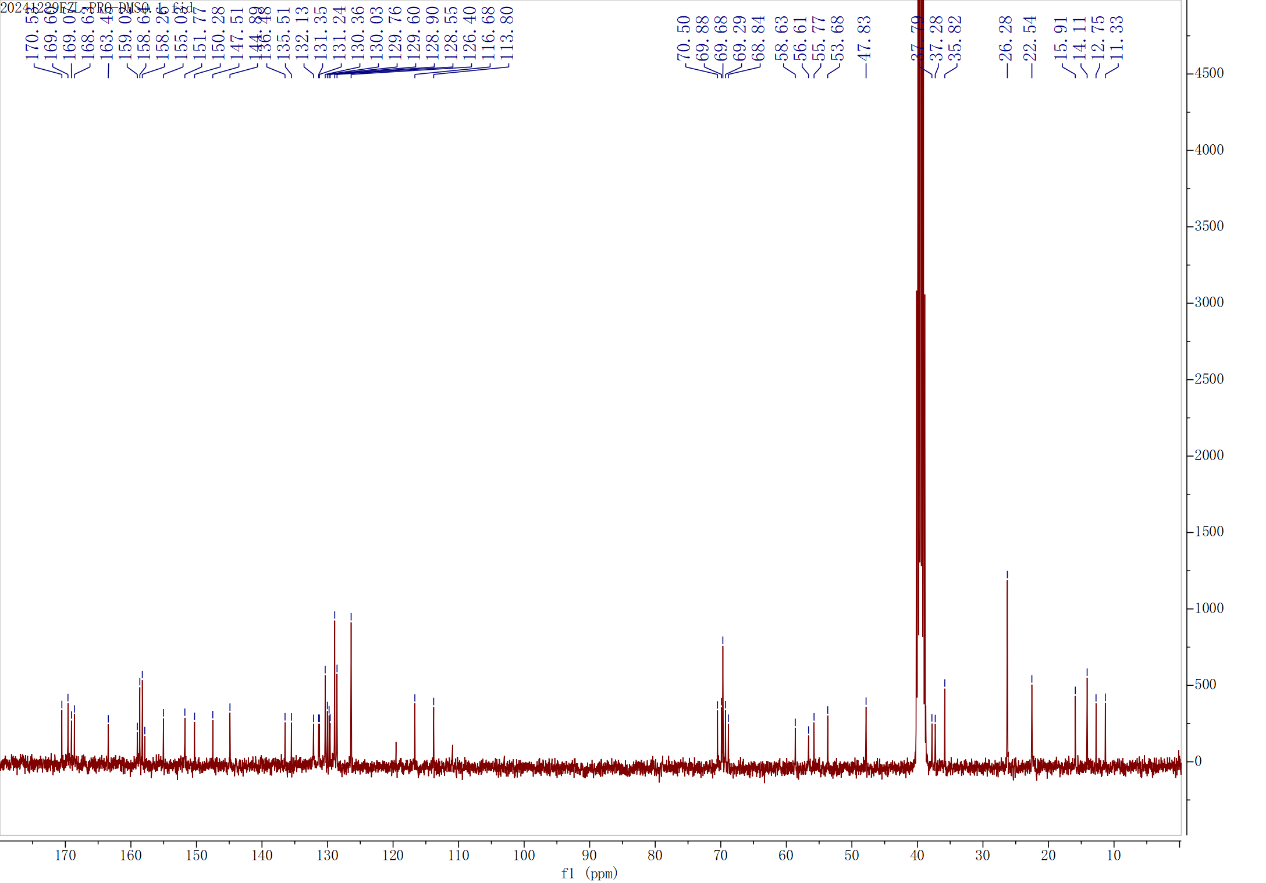


**Figure S9** ^13^C NMR spectrum of PRO.

**Figure S10** HRMS spectrum of compound **5**.


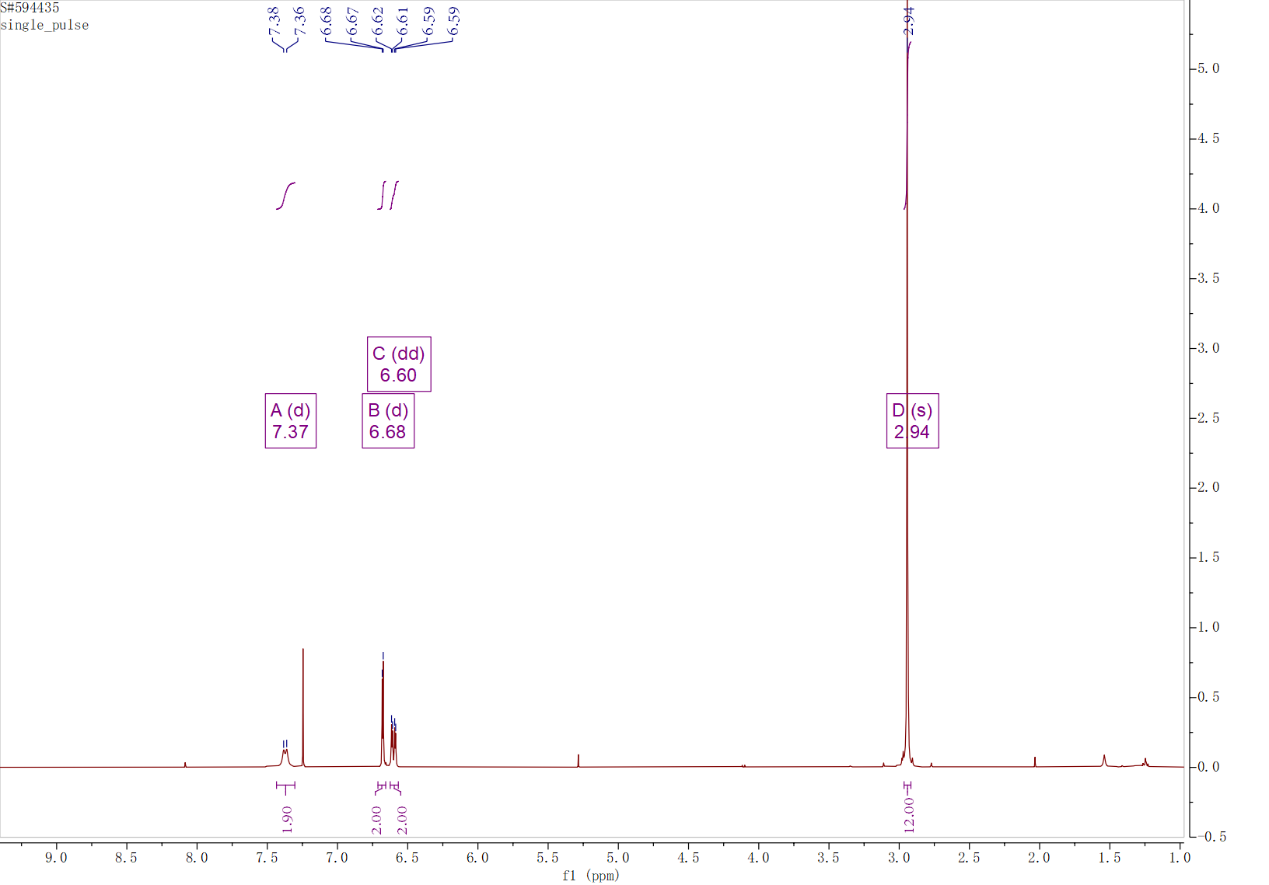


**Figure S11** ^1^H NMR spectrum of compound **5**.


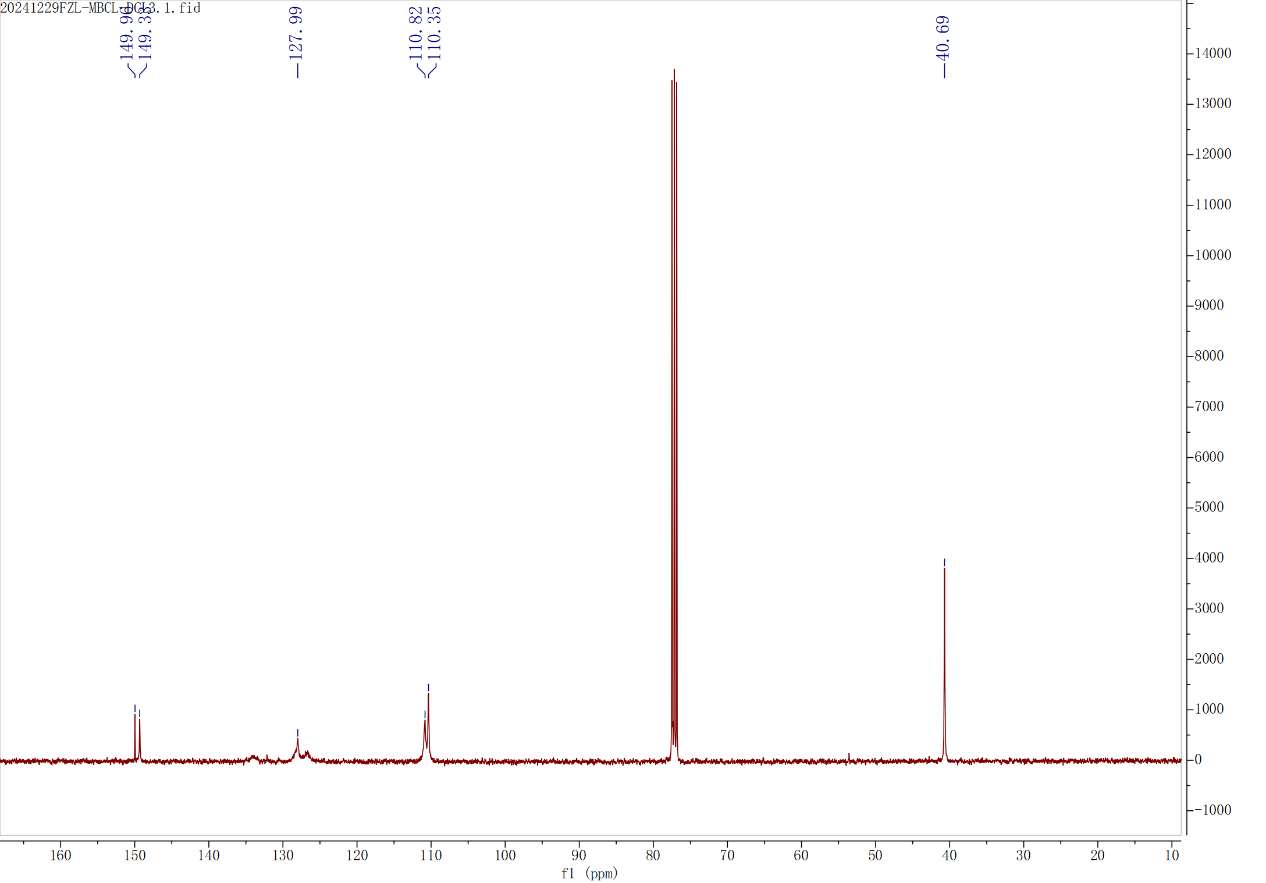


**Figure S12** ^13^C NMR spectrum of compound **5**.

**Figure S13** HRMS spectrum of compound **6**.


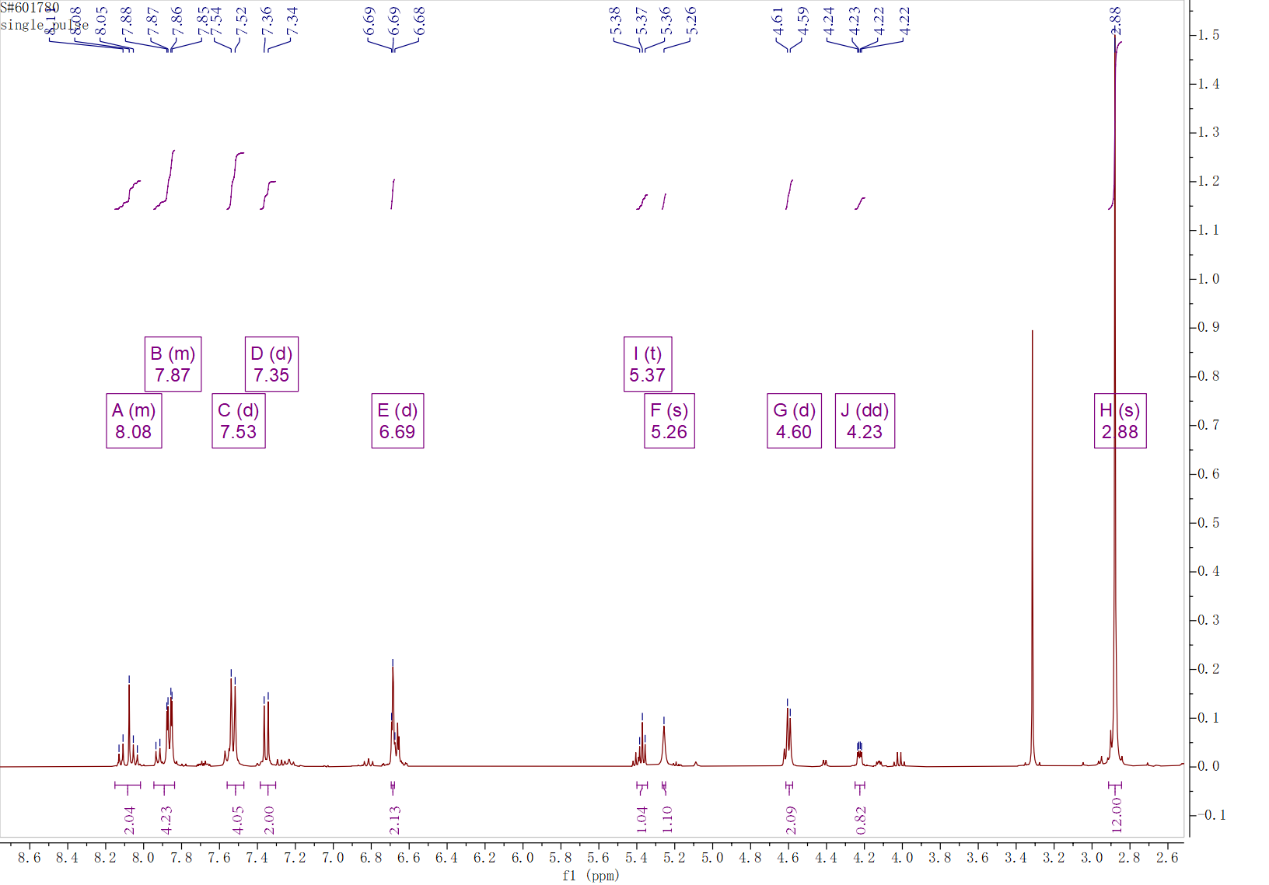


**Figure S14** ^1^H NMR spectrum of compound **6**.


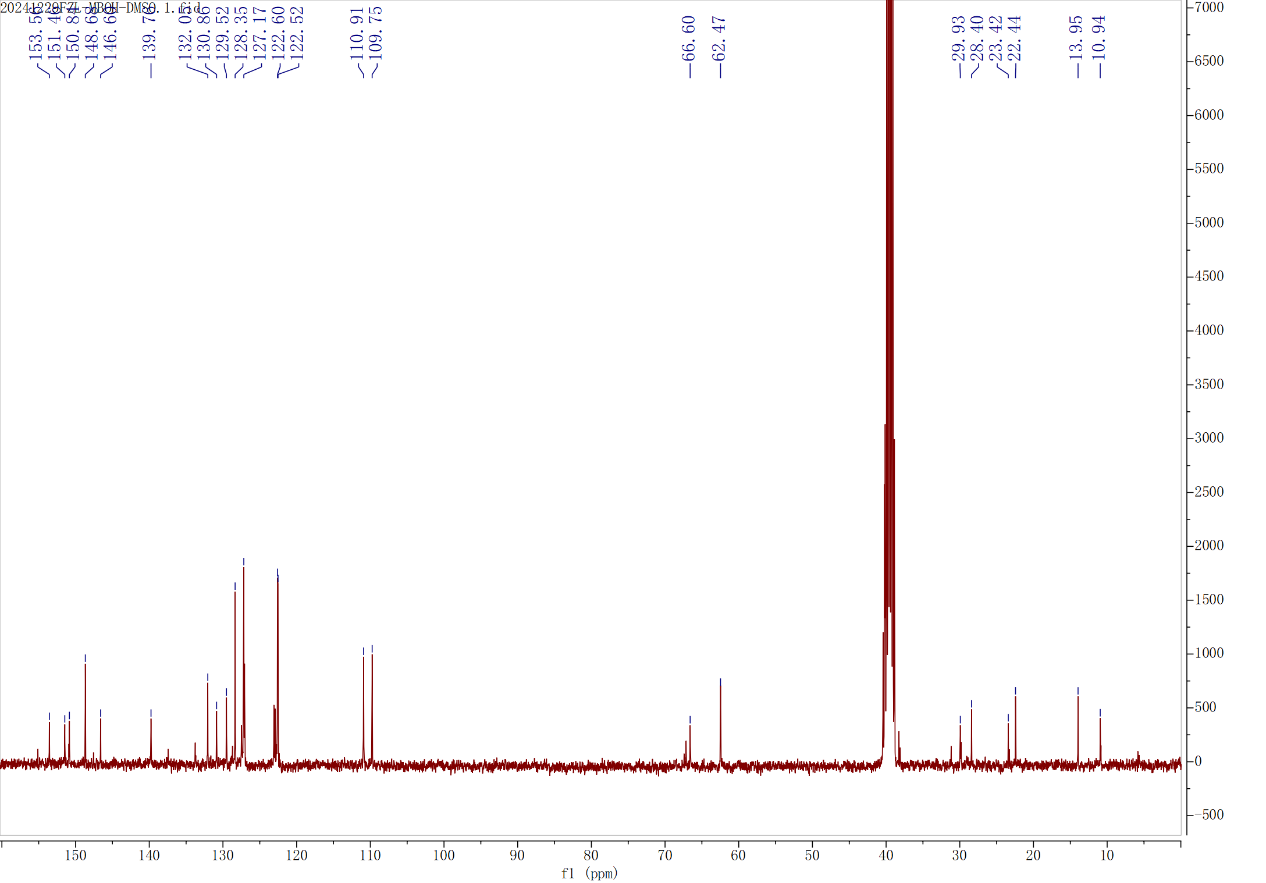


**Figure S15** ^13^C NMR spectrum of compound **6**.

**Figure S16** HRMS spectrum of compound **7**.


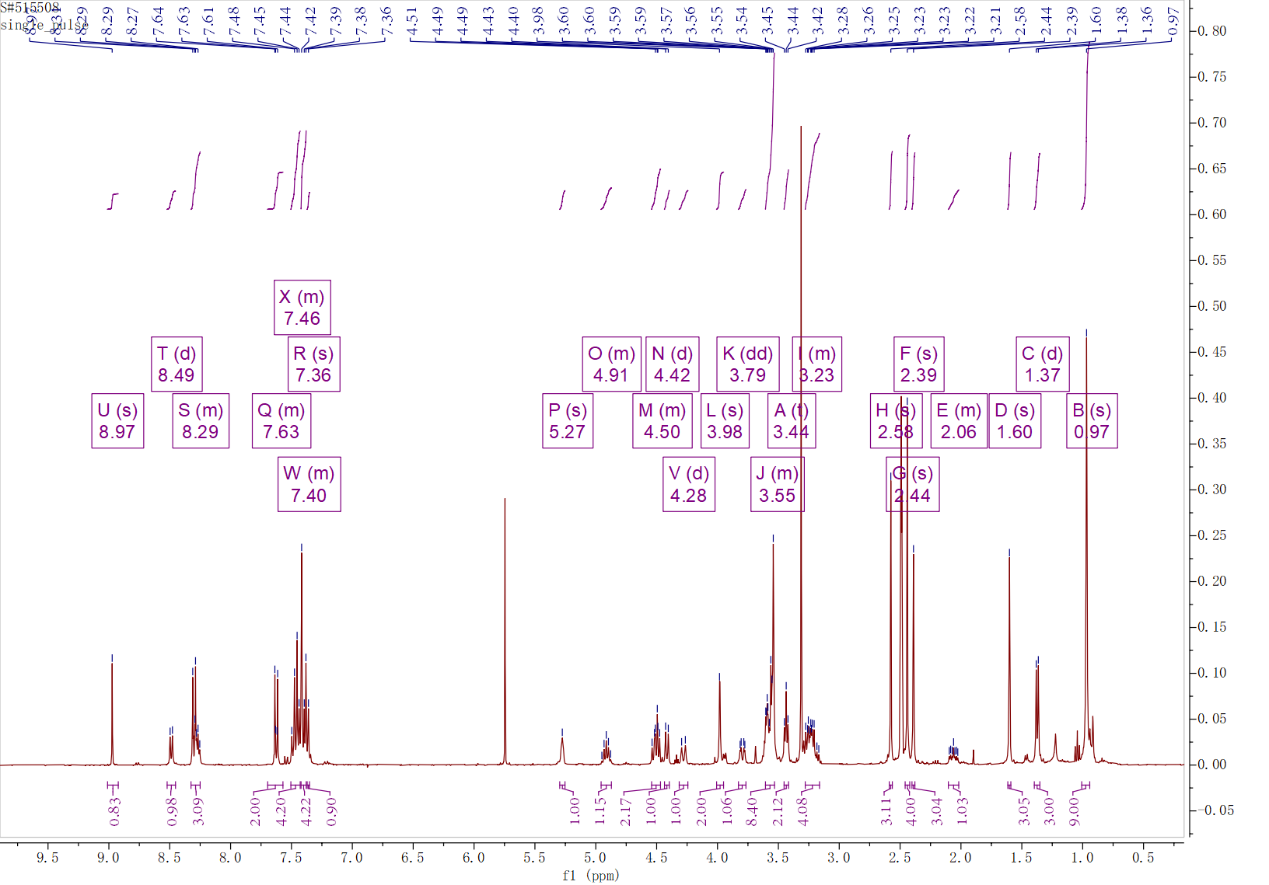


**Figure S17** ^1^H NMR spectrum of compound **7**.


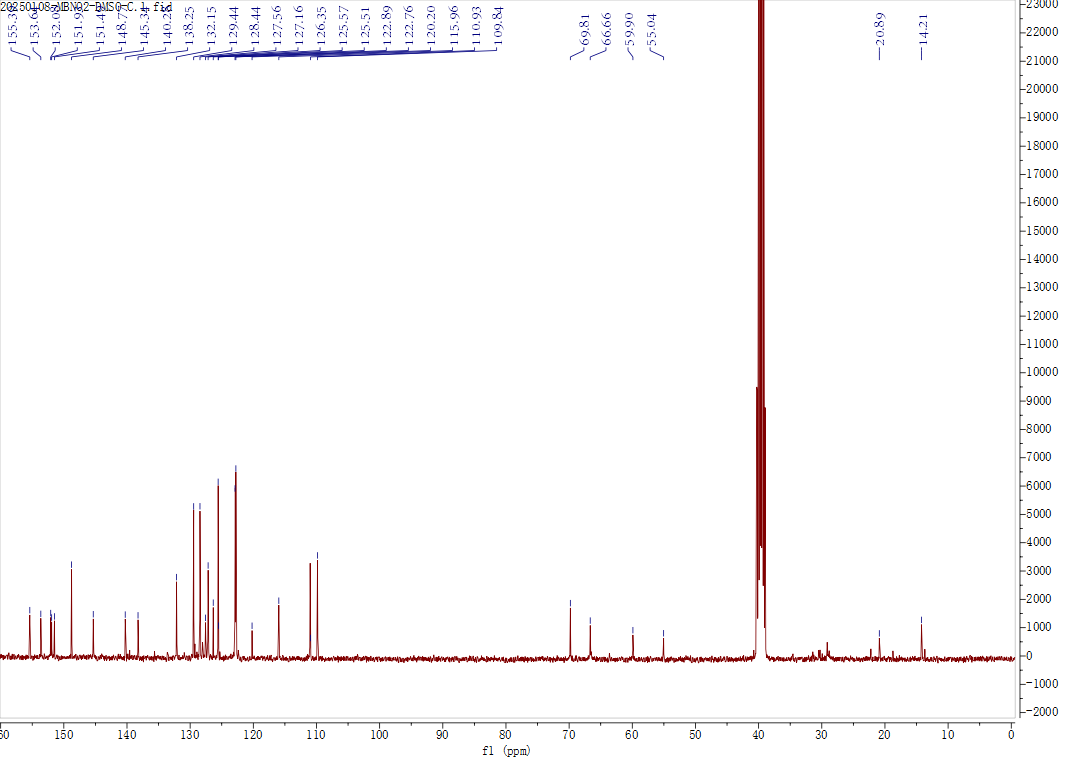


**Figure S18** ^13^C NMR spectrum of compound **7**.

**Figure S19** HRMS spectrum of AZO-PRO.


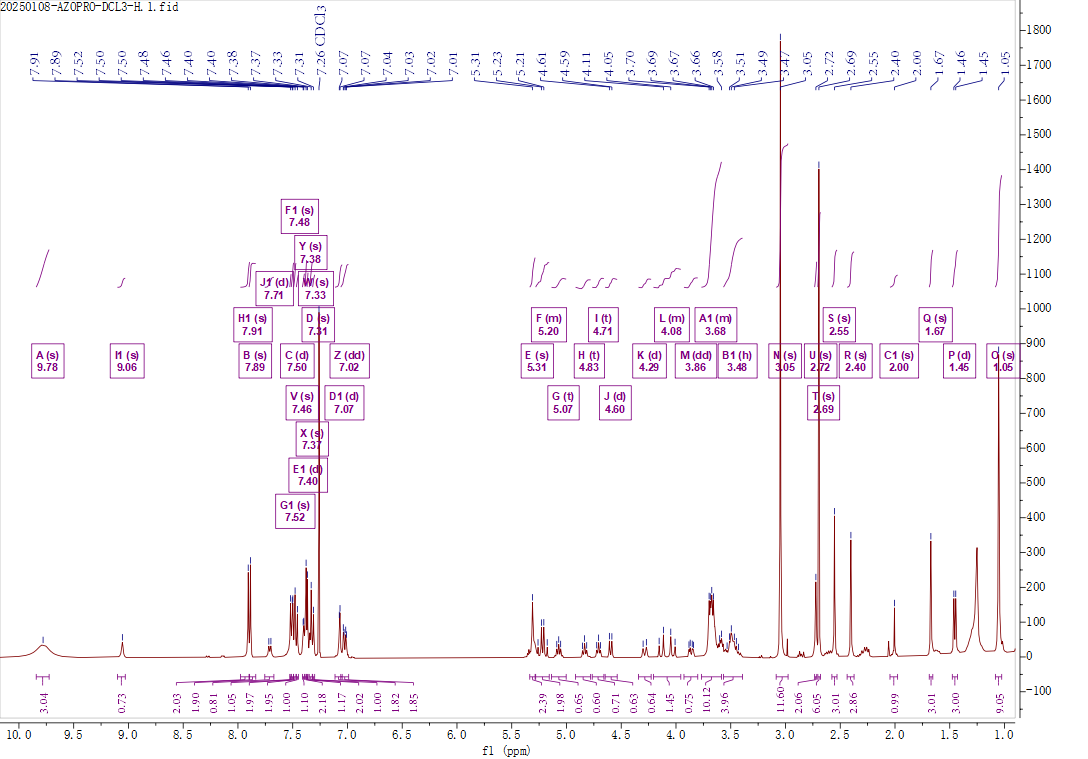


**Figure S20** ^1^H NMR spectrum of AZO-PRO.


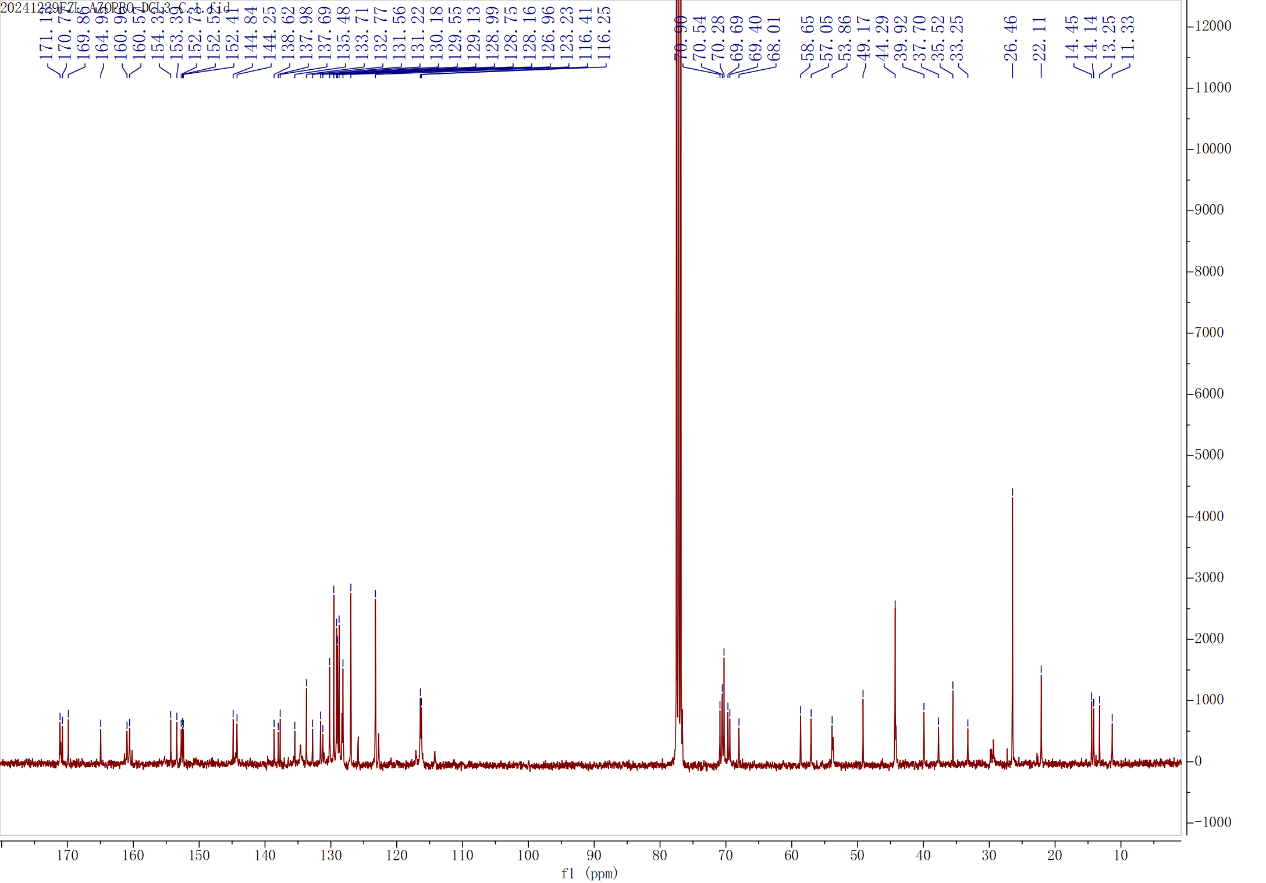


**Figure S21** ^13^C NMR spectrum of AZO-PRO.


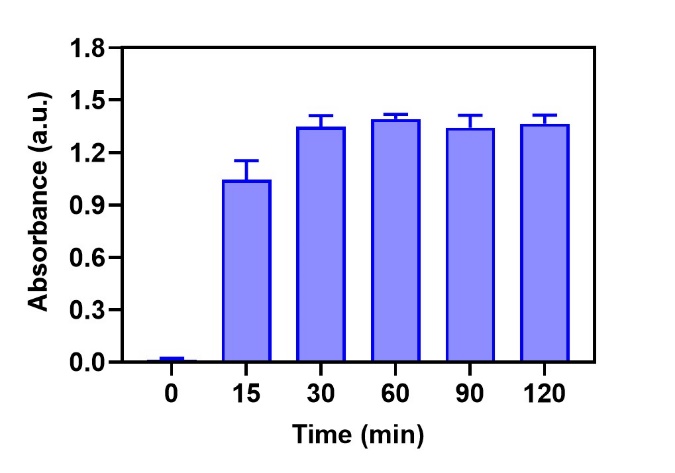


**Figure S22** The UV absorption intensity changed after incubation of AZO-PRO (50 μmol/L) with Na_2_S_2_O_4_ (50 mmol/L) in PBS buffer (5 mmol/L, *V*_Acetonitrile_: *V*_PBS_ =1: 1) at 37 °C for different times. Data are presented as mean ± SD (*n* = 6).


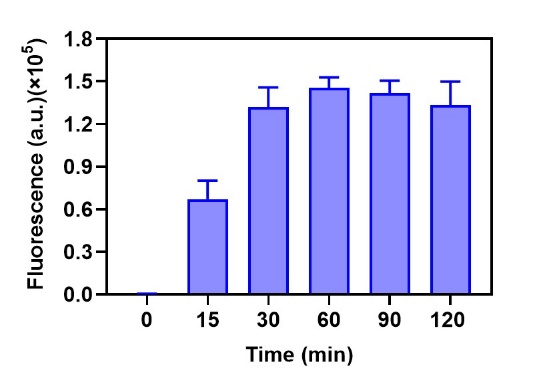


**Figure S23** Fluorescence intensity of AZO-PRO (50 μmol/L) and Na_2_S_2_O_4_ (50 mmol/L) after various incubation periods. Ex = 620 nm, Em = 690 nm. Data are presented as mean ± SD (*n* = 6).


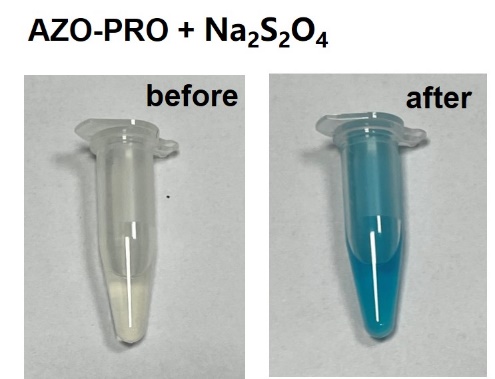


**Figure S24** Photographs of color changes before (left) and after (right) activation of the hypoxia-activated prodrug AZO-PRO.


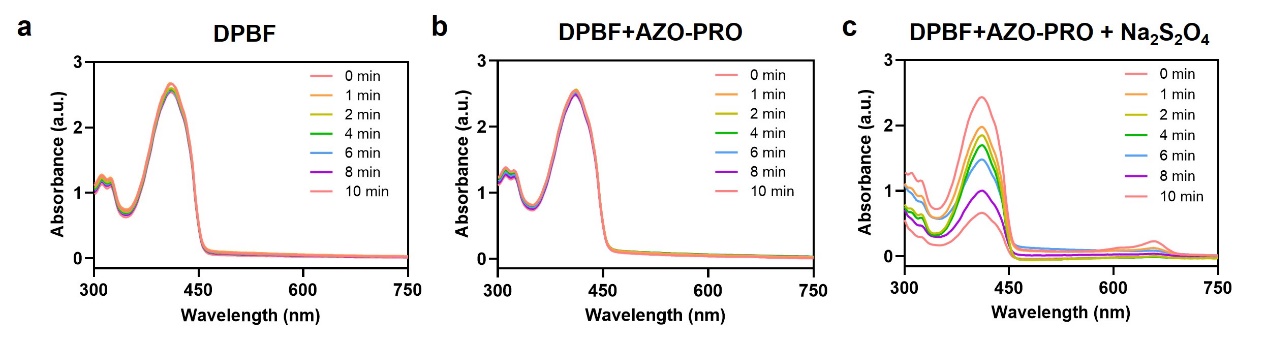


**Figure S25** UV absorption spectra of (A)DPBF (60 μmol/L), (B)AZO-PRO (5 μmol/L), and (C) AZO-PRO (5 μmol/L) +Na_2_S_2_O_4_ (25 mmol/L) +DPBF (60 μmol/L) irradiated with 660 nm laser for different times.


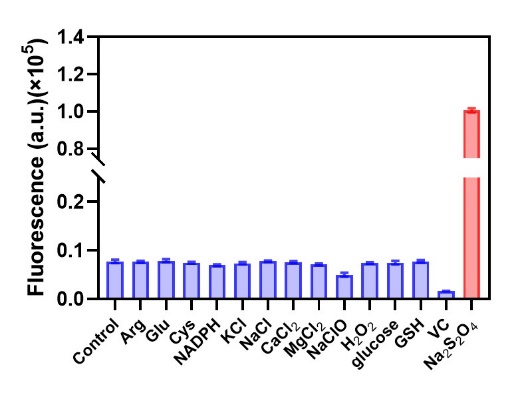


**Figure S26** Fluorescence signal (690 nm) of AZO-PRO after incubation with various biological species in respective buffers at 37 °C for 2 h. Data are presented as mean ± SD (*n* = 3).


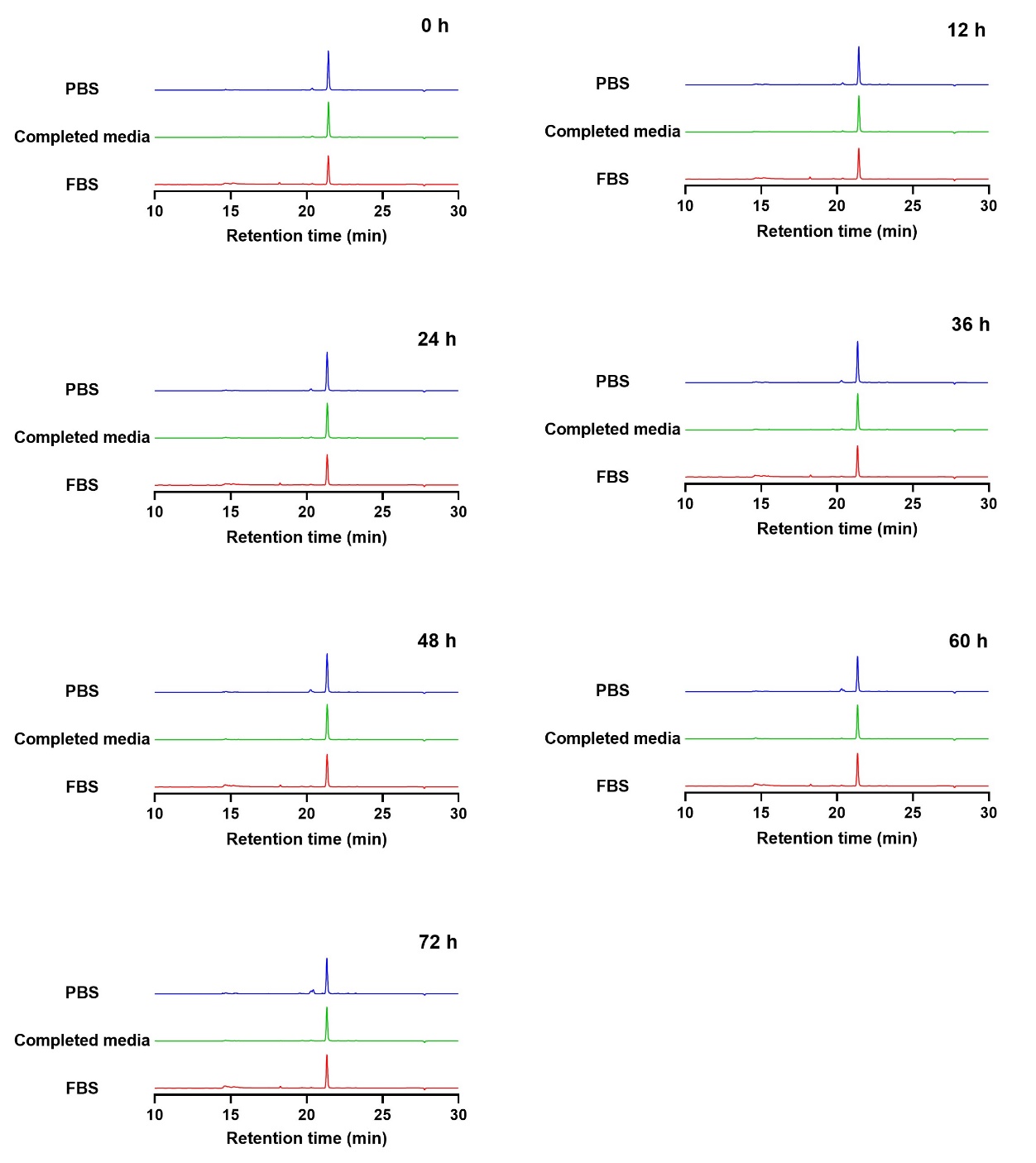


**Figure S27** Stability analysis of AZO-PRO in PBS, fetal bovine serum, and complete media (containing 10% fetal bovine serum in DMEM). HPLC analysis of AZO-PRO at 0, 12, 24, 36 h, 48, 60 and 72 h after dilution to a final concentration of 100 μmol/L in different solvents containing 1% *v*/*v* DMSO.


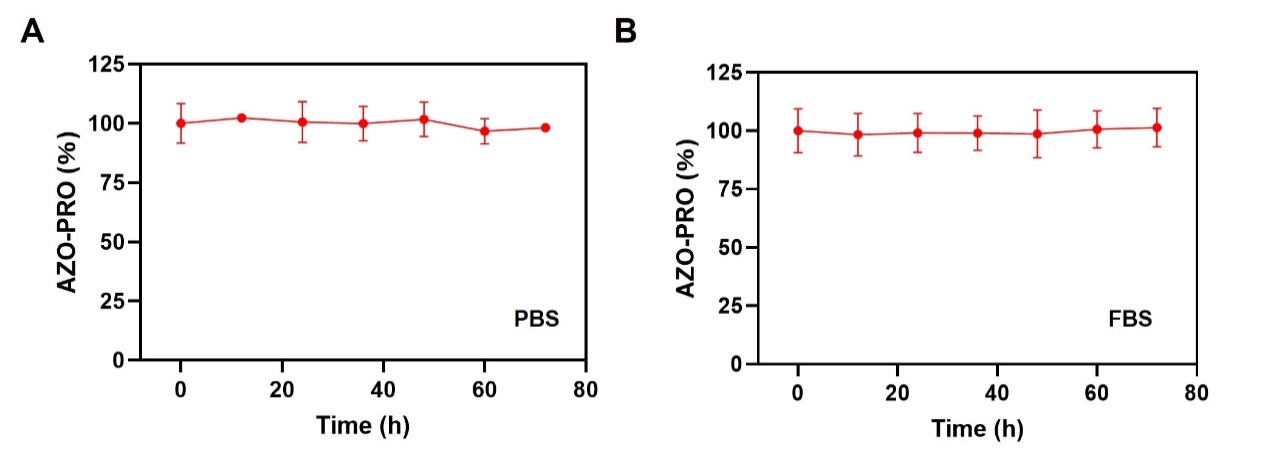


**Figure S28** Normalized concentration changes of AZO-PRO in (A) PBS and (B) fetal calf serum (FBS) over time.


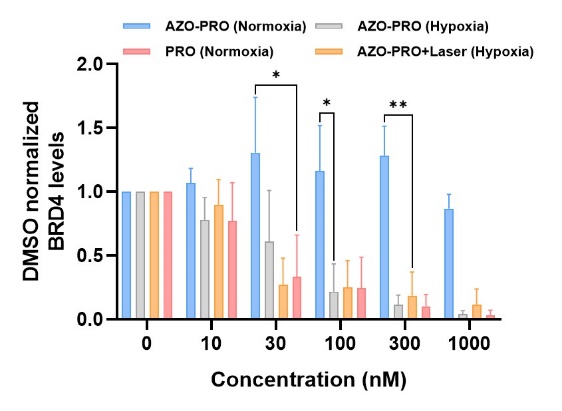


**Figure S29** Quantification of BRD4 protein levels was calculated relative to DMSO control. Data are presented as mean ± SD *n* = 3. **P*<0.05, ***P*<0.01.


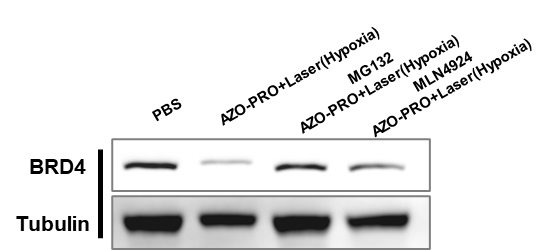


**Figure S30** Western blotting analysis of BRD4 levels in MCF-7 cells treated with the 100 nmol/L AZO-PRO + Laser (Hypoxia) for 24 h. For the proteasome inhibitor experiments, cells were pretreated with 10 μmol/L MG132 or 1 μmol/L MLN4924.


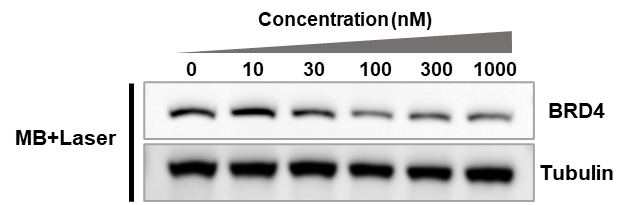


**Figure S31** Western blotting analysis of BRD4 expression level in MCF-7 cells treated with different concentrations of MB for 24 h.


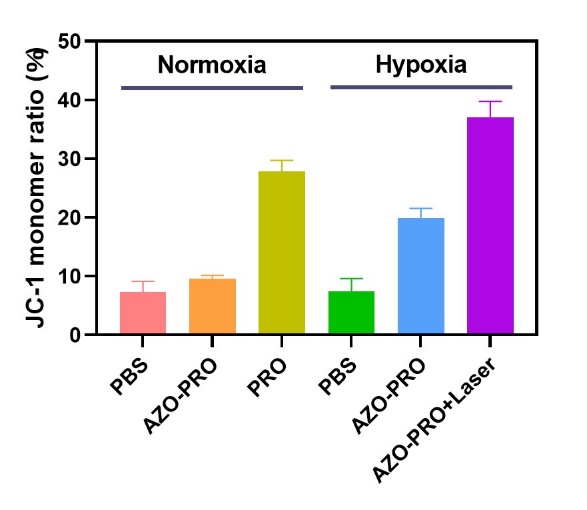


**Figure S32** The statistical analysis on the JC-1 monomer ratio in MCF-7 cells with different treatments. Data are presented as mean ± SD *n* = 3.


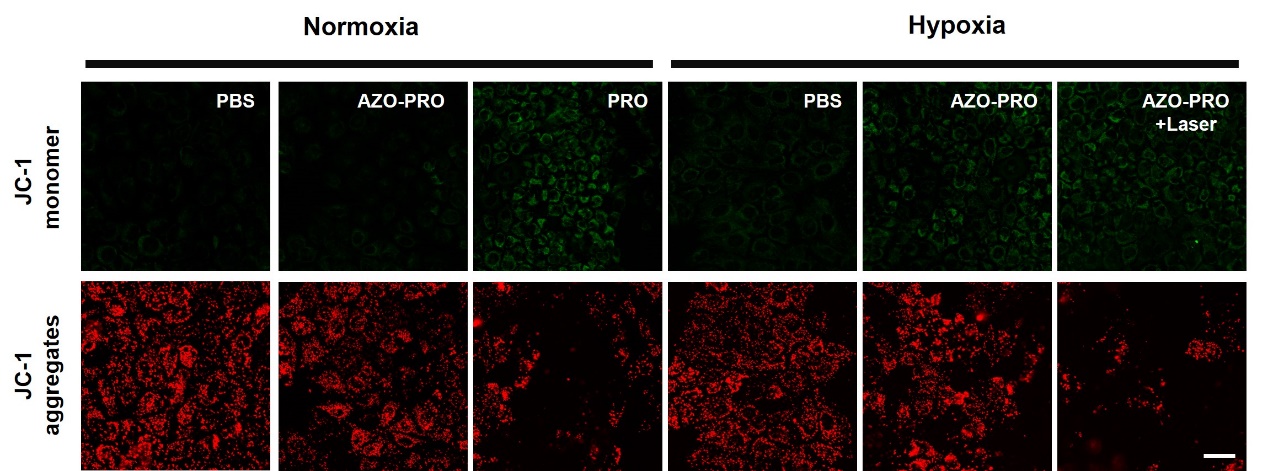


**Figure S33** JC-1 staining of MCF-7 cells with different treatments. Scale bar = 50 μm. green: J-monomer, red: J-aggregates.


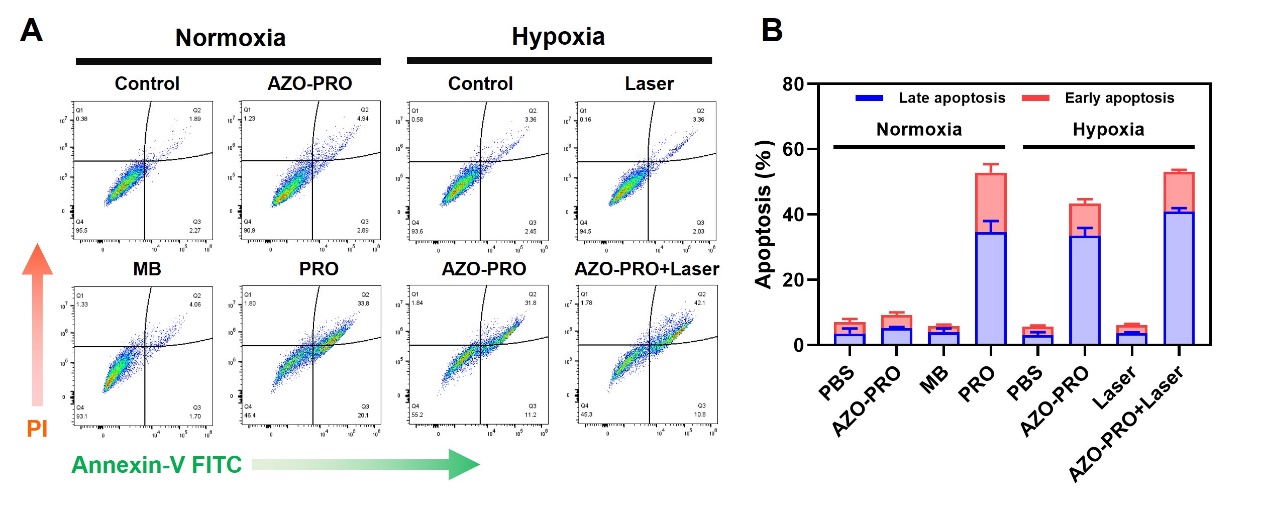


**Figure S34** (A) Flow cytometry was used to analyze the apoptosis of MCF-7 cells treated with different drugs. (B) The quantitative analysis of the corresponding cell apoptosis percentage. Data are presented as mean ± SD (*n* = 3).


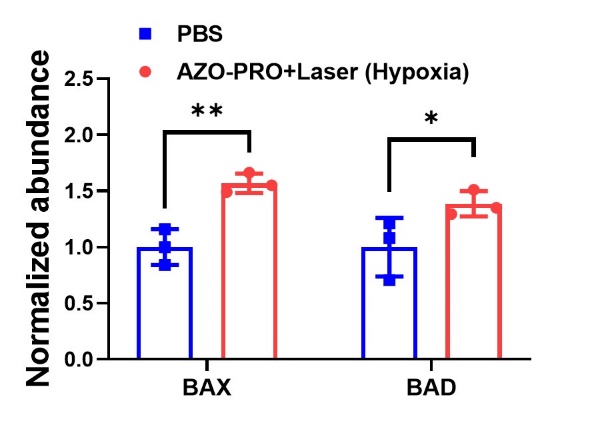


**Figure S35** Quantification of representative BAX and BAD proteins of mass spectrometry proteomics. Data are presented as mean ± SD; **P*<0.05, ***P*<0.01.


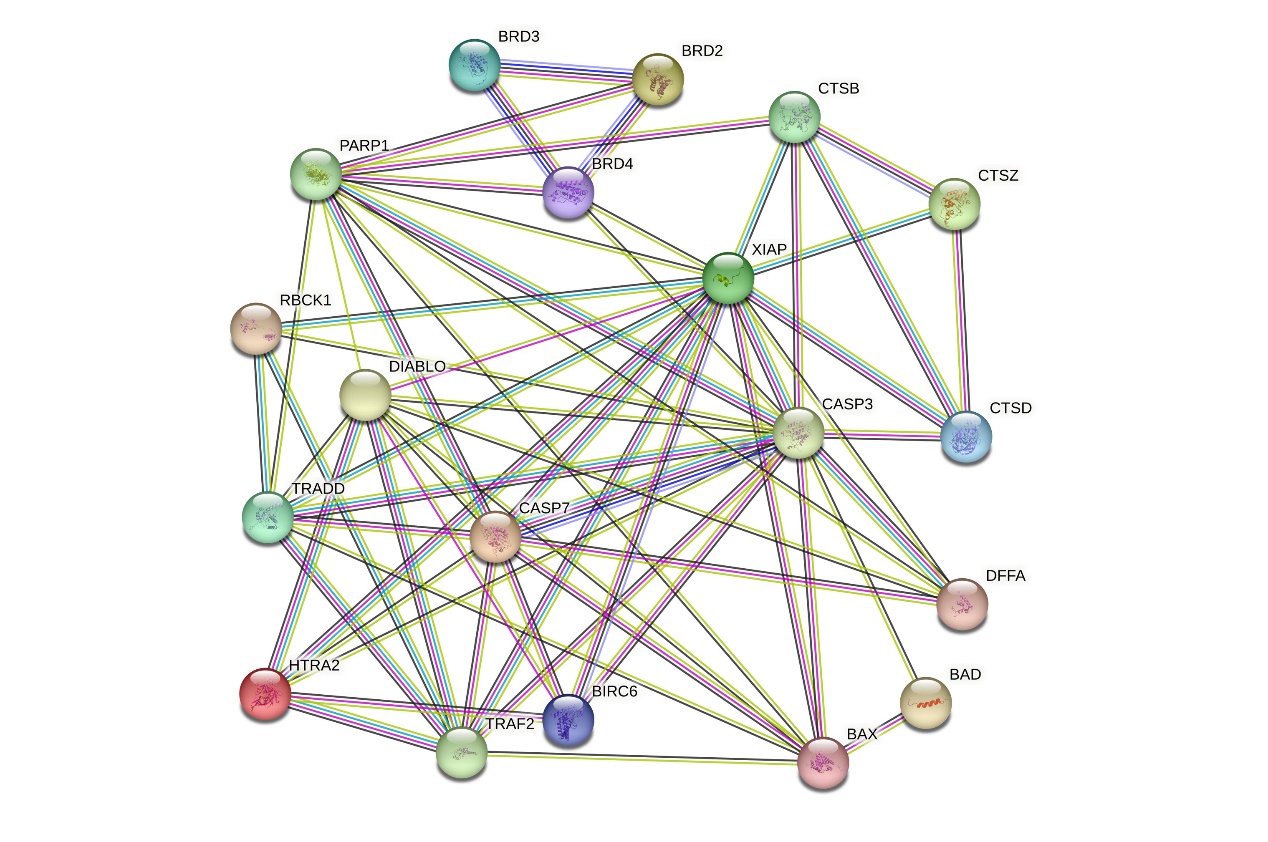


**Figure S36** Protein-Protein Interaction Network of 19 proteins of interest. The picture was drawn by STRING. (STRING: <https://string-db.org/>).


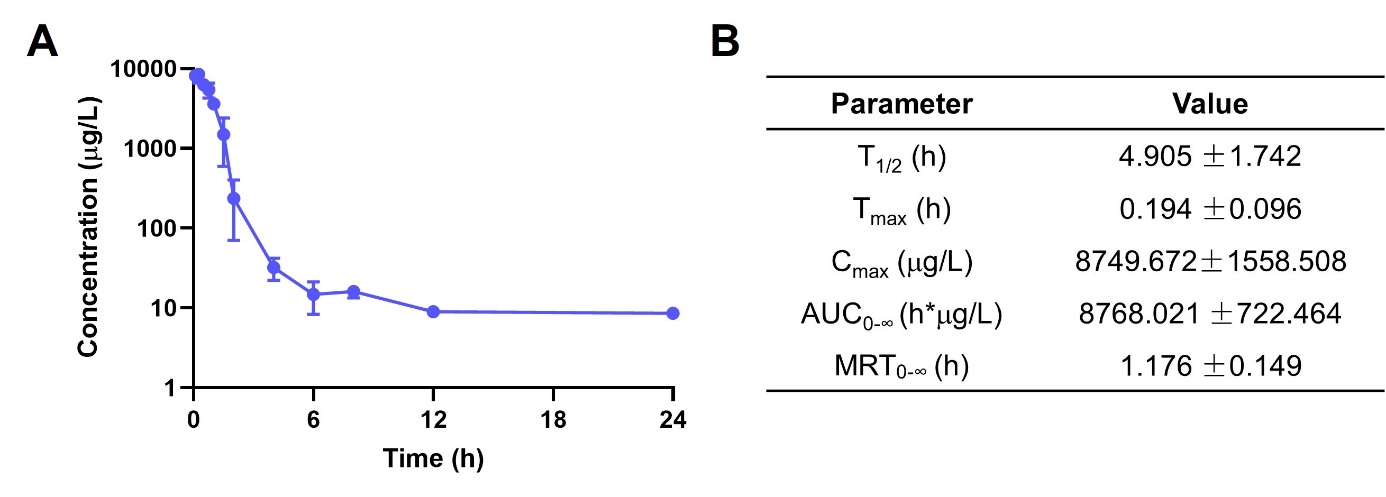


**Figure S37** (A) Plasma concentrations of AZO-PRO after 10 mg/kg intravenous injection over 24 h in mice. Data are presented as mean ± SD, *n* = 3. (B) Pharmacokinetics parameters of AZO-PRO in mice. Data are presented as mean ± SD, *n* = 3.


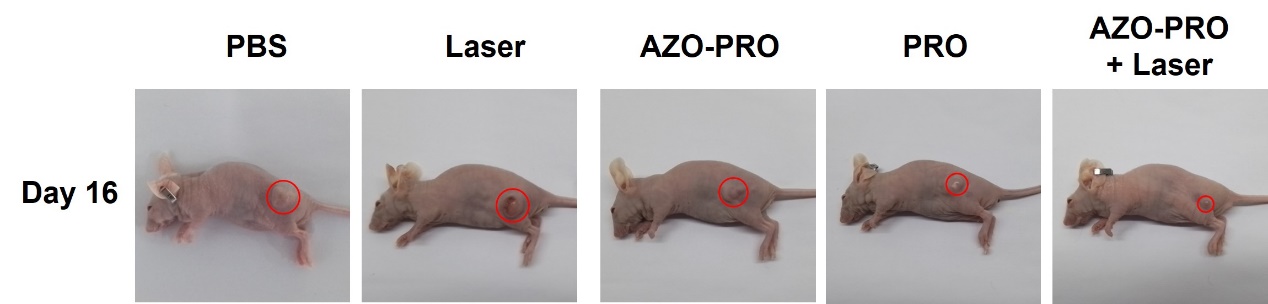


**Figure S38** Pictures of mice from different groups at Day 16 after different treatments.


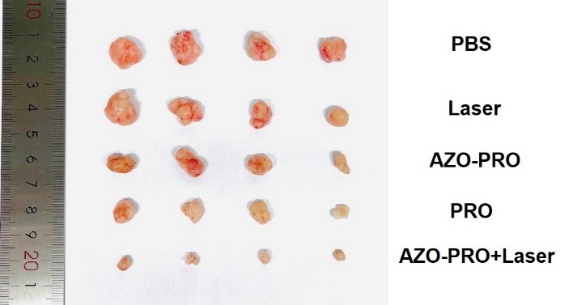


**Figure S39** Photographs of excised tumors.


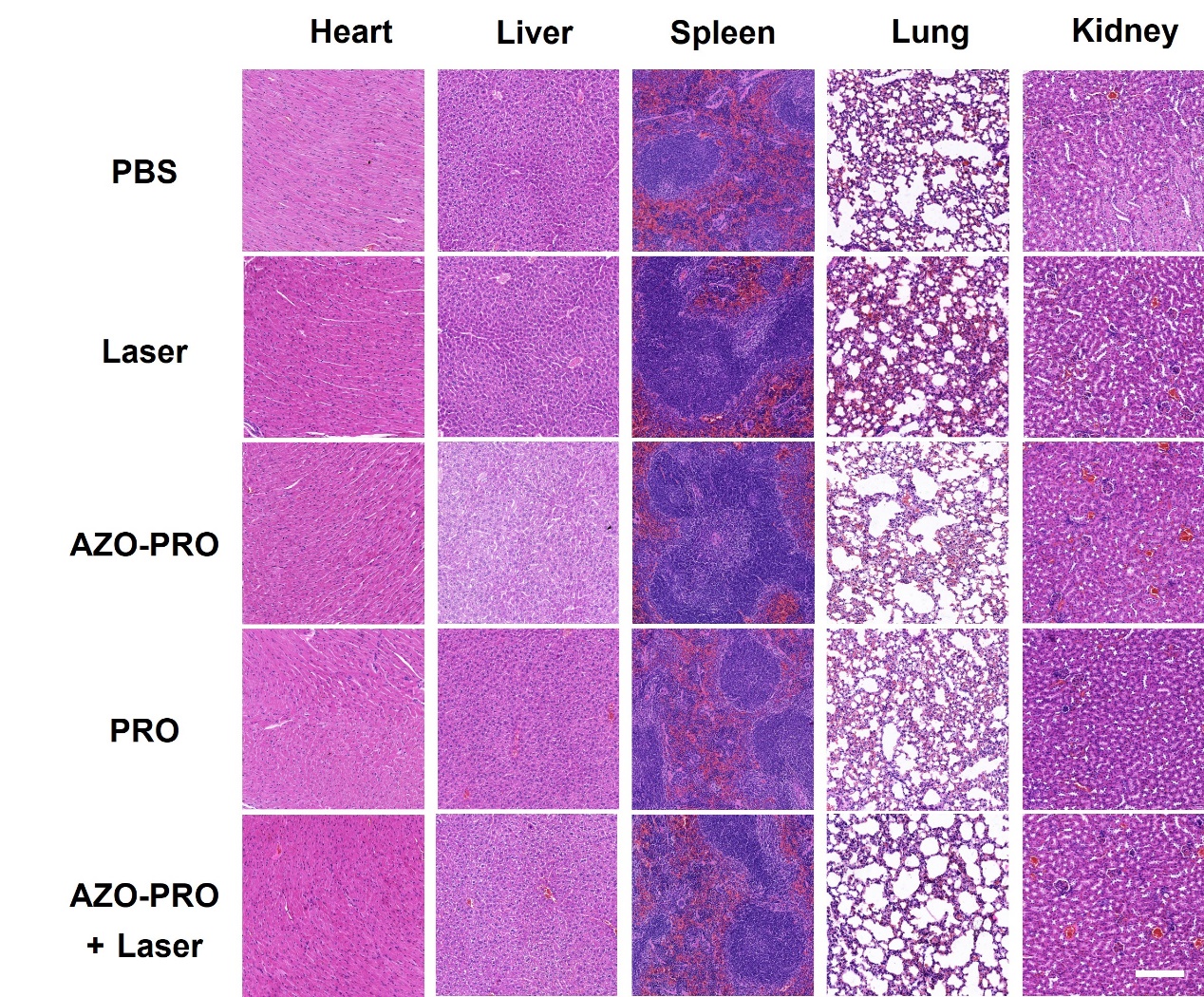


**Figure S40** Representative histological images of the major organs. The major organs of MCF-7 bearing mice receiving different treatments were collected and stained with hematoxylin-eosin (H&E) for histological analysis. Scale bar= 200 μm.


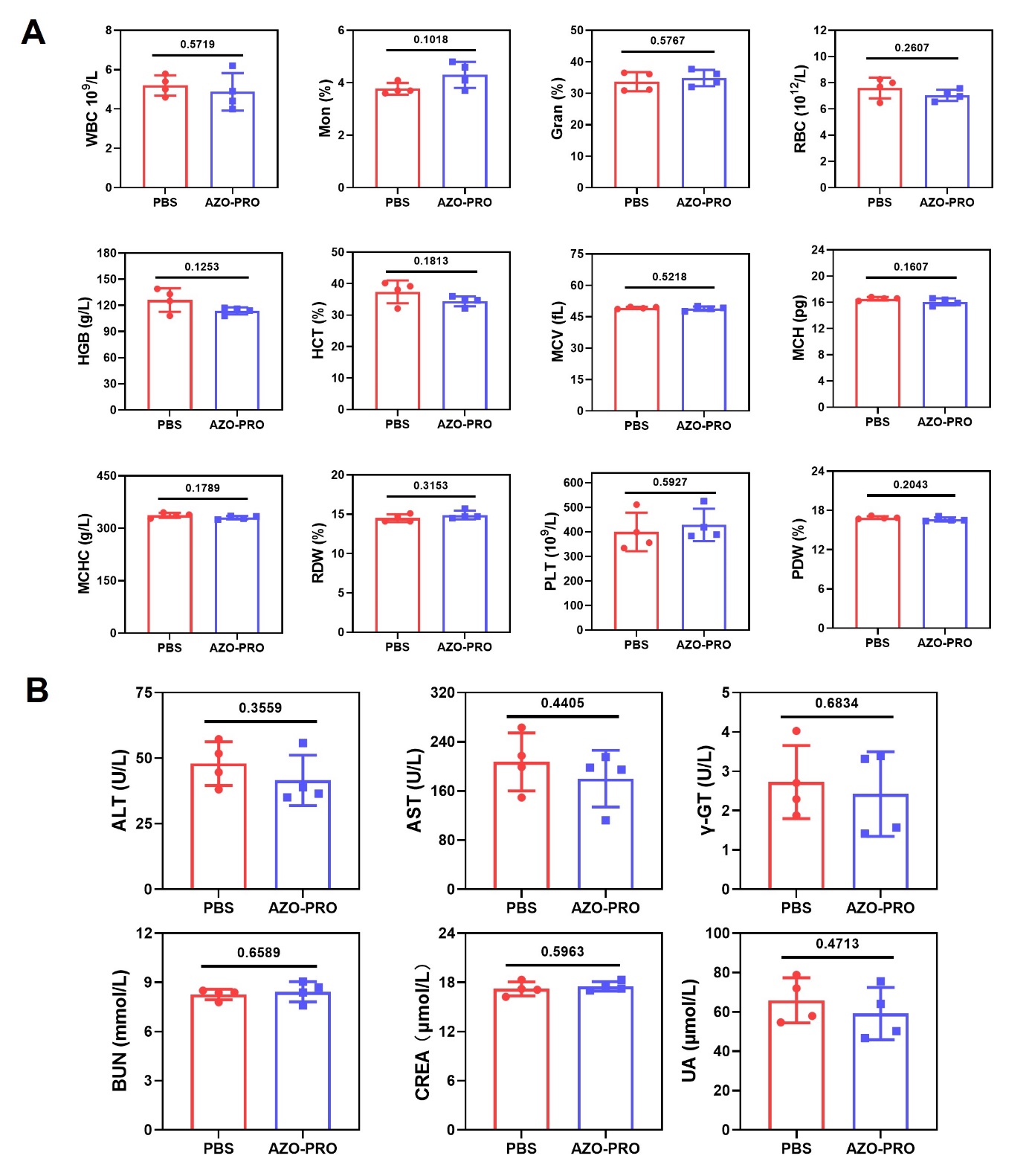


**Figure S41** (A) Blood biochemical indicators: white blood cells (WBC), monocytes percentage (Mon), neutrophil percentage (Gran), red blood cells (RBC), hemoglobin (HGB), hematocrit (HCT), mean corpuscular volume (MCV), mean corpuscular hemoglobin (MCH), mean corpuscular hemoglobin concentration (MCHC), red cell distribution width (RDW), platelet (PLT), and platelet distribution width (PDW). Data are presented as mean ± SD, *n* = 4. (B) Liver and kidney function indicators: alanine aminotransferase (ALT), aspartate aminotransferase (AST), Gamma-glutamyltransferase (*γ*-GT), urea (UREA) creatinine (CREA) and uric acid (UA). Data are presented as mean ± SD, *n* = 4.


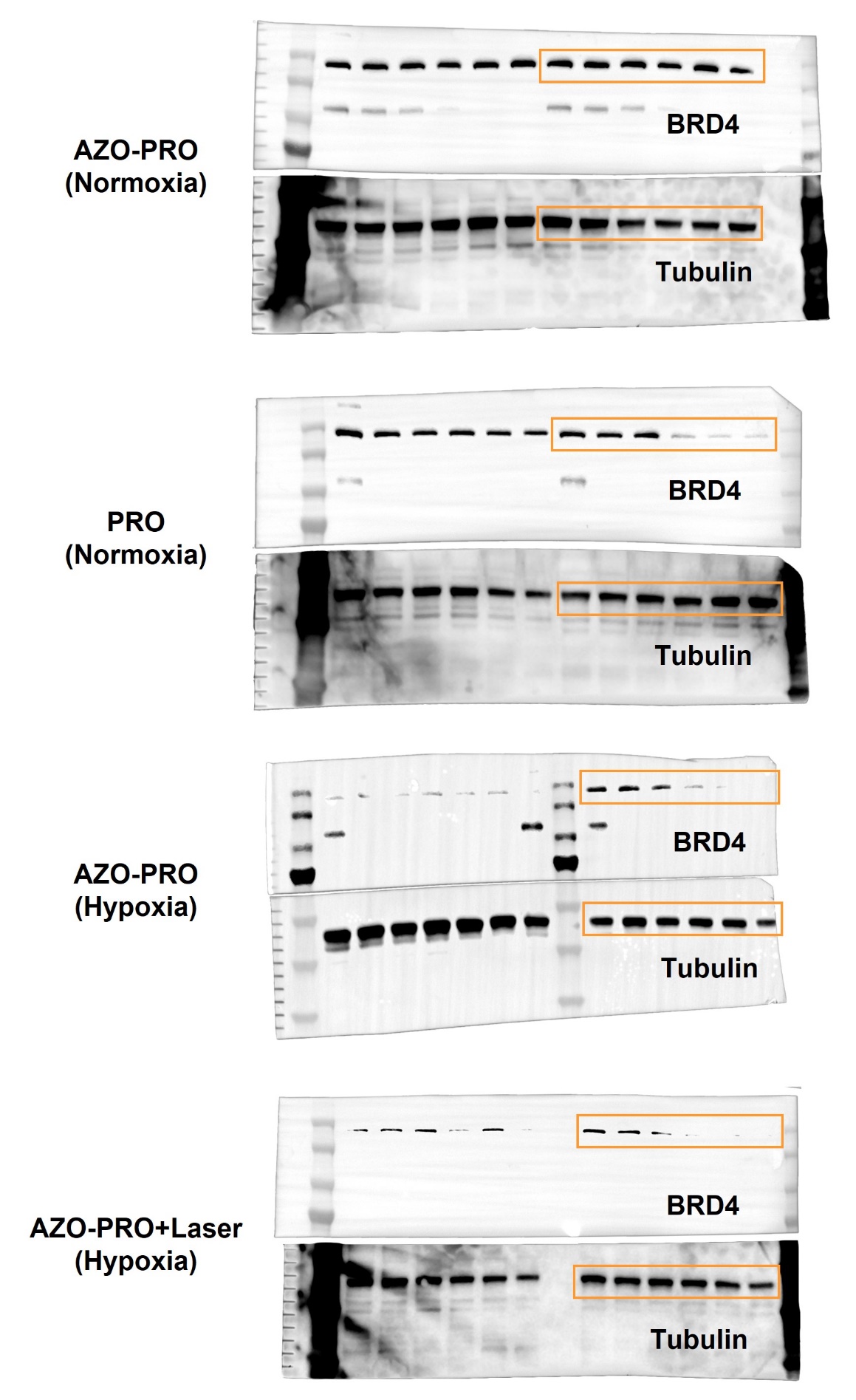


**Figure S42** Uncropped western blot source data for Fig. 4a.


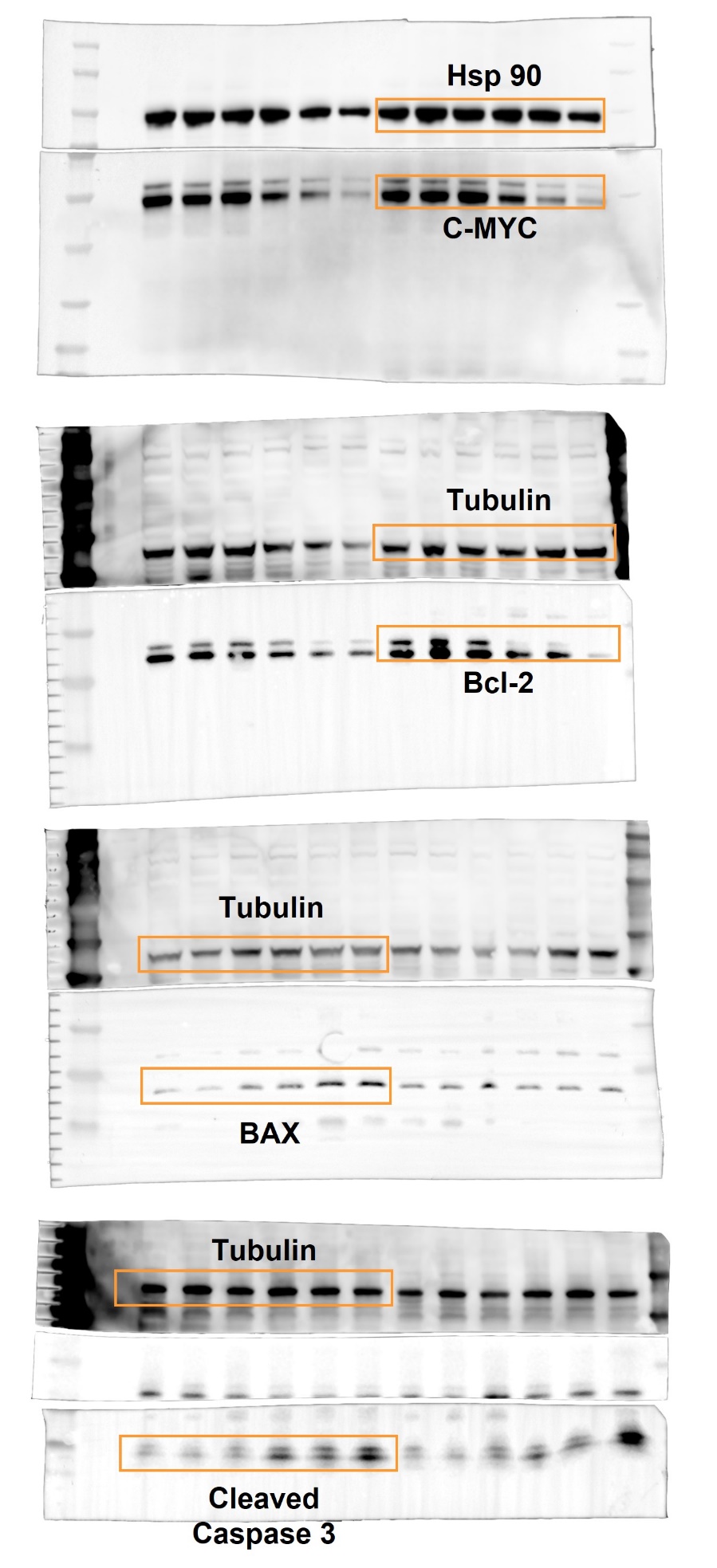


**Figure S43** Uncropped western blot source data for Fig. 5b.


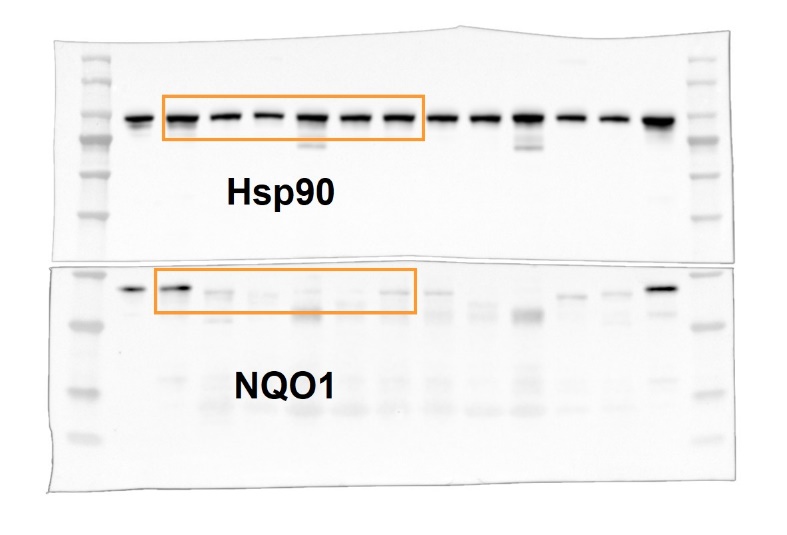


**Figure S44** Uncropped western blot source data for Figure 6g.
